# Neuromechanics and Energetics of Walking With an Ankle Exoskeleton Using Neuromuscular-Model Based Control: A Parameter Study

**DOI:** 10.3389/fbioe.2021.615358

**Published:** 2021-04-09

**Authors:** Benjamin A. Shafer, Sasha A. Philius, Richard W. Nuckols, James McCall, Aaron J. Young, Gregory S. Sawicki

**Affiliations:** ^1^George W. Woodruff School of Mechanical Engineering, Georgia Institute of Technology, Atlanta, GA, United States; ^2^Institute for Robotics and Intelligent Machines, Georgia Institute of Technology, Atlanta, GA, United States; ^3^Joint Department of Biomedical Engineering, North Carolina State University and University of North Carolina at Chapel Hill, Raleigh, NC, United States; ^4^Harvard John A. Paulson School of Engineering and Applied Sciences, Harvard University, Boston, MA, United States

**Keywords:** powered ankle exoskeleton, neuromuscular model, locomotion neuromechanics, human walking, muscle electromyography, metabolic energy cost, plantarflexor muscle-tendon mechanics

## Abstract

Powered ankle exoskeletons that apply assistive torques with optimized timing and magnitude can reduce metabolic cost by ∼10% compared to normal walking. However, finding individualized optimal control parameters is time consuming and must be done independently for different walking modes (e.g., speeds, slopes). Thus, there is a need for exoskeleton controllers that are capable of continuously adapting torque assistance in concert with changing locomotor demands. One option is to use a biologically inspired, model-based control scheme that can capture the adaptive behavior of the human plantarflexors during natural gait. Here, based on previously demonstrated success in a powered ankle-foot prosthesis, we developed an ankle exoskeleton controller that uses a neuromuscular model (NMM) comprised of a Hill type musculotendon driven by a simple positive force feedback reflex loop. To examine the effects of NMM reflex parameter settings on (i) ankle exoskeleton mechanical performance and (ii) users’ physiological response, we recruited nine healthy, young adults to walk on a treadmill at a fixed speed of 1.25 m/s while donning bilateral tethered robotic ankle exoskeletons. To quantify exoskeleton mechanics, we measured exoskeleton torque and power output across a range of NMM controller Gain (0.8–2.0) and Delay (10–40 ms) settings, as well as a High Gain/High Delay (2.0/40 ms) combination. To quantify users’ physiological response, we compared joint kinematics and kinetics, ankle muscle electromyography and metabolic rate between powered and unpowered/zero-torque conditions. Increasing NMM controller reflex Gain caused increases in average ankle exoskeleton torque and net power output, while increasing NMM controller reflex Delay caused a decrease in net ankle exoskeleton power output. Despite systematic reduction in users’ average biological ankle moment with exoskeleton mechanical assistance, we found no NMM controller Gain or Delay settings that yielded changes in metabolic rate. *Post hoc* analyses revealed weak association at best between exoskeleton and biological mechanics and changes in users’ metabolic rate. Instead, changes in users’ summed ankle joint muscle activity with powered assistance correlated with changes in their metabolic energy use, highlighting the potential to utilize muscle electromyography as a target for on-line optimization in next generation adaptive exoskeleton controllers.

## Introduction

Lower-limb exoskeletons are a promising approach to reduce human effort by providing mechanical assistance to restore, replace, or augment the function of biological musculotendons during walking (Sawicki et al., 2020). Analysis of human gait biomechanics provides a roadmap that can be used to guide the location (e.g., ankle, knee or hip), timing and magnitude of mechanical assistance applied by an exoskeleton system. Indeed, based on the large contribution of ankle musculotendons to the overall mechanical power generated by the lower-limb during walking (Farris and Sawicki, 2012), researchers and engineers have focused heavily on delivering power with ankle exoskeletons as a means for reducing metabolic cost of walking (Sawicki and Ferris, 2008; Malcolm et al., 2013; Mooney et al., 2014; Jackson and Collins, 2015; Galle et al., 2017; Zhang et al., 2017b; Grimmer et al., 2019). These studies clearly demonstrate that powered ankle exoskeletons are a viable means to decrease metabolic cost of walking, in the best case, by ∼10% when compared to walking in normal shoes (Galle et al., 2017). Although the number of ankle exoskeletons demonstrating metabolic benefits in a controlled laboratory setting continues to grow (Sawicki et al., 2020), to be useful in the real-world, these devices need to be able to automatically adjust to both the user and the environment.

A major factor in determining exoskeleton performance is the control architecture that is used to generate the commands to the motors that apply torques to the lower-limb joints (Cain et al., 2007; Koller et al., 2017; Kirby, 2018). A wide variety of torque control schemes have been demonstrated in lower-limb wearable robots (Jiménez-Fabián and Verlinden, 2012) with a subset employed in powered ankle exoskeletons (Zhang et al., 2015, 2017a). Some common examples include applying preset stiffness and damping values set as a function of joint angle (Nuckols and Sawicki, 2020) or stride percentage (i.e., impedance control); directly driving the exoskeleton actuators with muscle activity of the user (i.e., myoelectric control) (Ferris et al., 2005, 2006; Fleischer et al., 2006; Sawicki and Ferris, 2008; Koller et al., 2015; Jackson and Collins, 2019) or specifying a set torque-time trajectory over the stride (Malcolm et al., 2013; Jackson and Collins, 2015; Galle et al., 2017; Zhang et al., 2017b). There are practical benefits and drawbacks to each of these control schemes, but they all share a common pitfall: reliance on *a priori* tuning of parameters. Tuning typically involves a time-consuming, exhaustive sweep of all combinations of the control parameters or, more recently, human-in-the-loop optimization (Zhang et al., 2017b) to find the set that is optimal for a given user and a chosen locomotion mode and outcome measure (e.g., the set that minimizes metabolic rate during walking at 1.25 m/s for user A). Even if an optimal parameter set is discovered under those unique conditions, they likely will not transfer to other locomotor modes that reflect normal walking behavior in the “real-world” (Orendurff et al., 2008). Using an adaptive controller that does not need to be tuned for each mode, optimally once per individual, could increase user acceptance of robotic exoskeletons for everyday use in dynamic environments.

Model-based ankle exoskeleton control is another option that may lead to robust, adaptive behavior in response to changes in the state of the user and/or the environment. In this control approach, a virtual muscle-tendon unit (MTU) is implemented to mimic the biological MTU. Typically, a Hill type MTU model (Zajac, 1989) is derived with contractile properties similar to the target biological analog. Then, the virtual MTU length change is driven by the user’s real-time joint kinematics through a virtual moment arm. The virtual muscle is stimulated by a modeled positive force feedback reflex pathway with a preset gain and delay to generate ongoing virtual muscle activation based on the previous force output of the model. In addition, the virtual muscle adheres to force-length and force-velocity relationships that can modify force output. Finally, the virtual MTU force is converted to an exoskeleton torque, again through the virtual moment arm. In essence, if the neuromuscular model were perfect, this approach would generate exoskeleton torque identical to the biological moment of the MTU targeted for assistance. In addition, because the assistance torque manifests from activation, length and velocity of a virtual muscle with a reflex pathway, the output should be able to spontaneously adapt to changing mechanical demands – similar to the biological system itself. Indeed, previous research has demonstrated that a reflex-driven, neuromuscular model (NMM) (Geyer and Herr, 2010) of the biological plantarflexors can provide robust torque commands to a powered ankle-foot prosthesis (Eilenberg et al., 2010) across a range of walking speeds and ground slopes without any need to adjust controller parameters per task. As a result, with NMM-controlled powered ankle-foot prostheses, amputees achieved normalized walking mechanics and energetics across a range of walking speeds (Markowitz et al., 2011; Herr and Grabowski, 2012). Recently, NMM-based control has been implemented on exoskeleton systems designed to restore movement to people with paralysis due to spinal cord injury or stroke (Wu et al., 2017; Durandau et al., 2019; Tamburella et al., 2020). However, to date, it is unclear whether NMM-based control is an effective strategy to provide assistive torques in parallel with neuromechanically intact ankle musculotendons and reduce effort during walking – even at a fixed-speed on level ground.

The purpose of this study was to implement an NMM-based controller designed to emulate the human ankle plantarflexors on a powered ankle exoskeleton and examine how it influences the neuromechanics and energetics of walking at a fixed-speed. Previous studies have demonstrated that both the timing and magnitude of ankle exoskeleton assistance are important for minimizing the metabolic cost of the user (Galle et al., 2017; Zhang et al., 2017b). Studies employing NMM-based control on powered ankle-foot prostheses have demonstrated that timing and magnitude of torque can be modulated by adjusting the Gain and Delay of the positive force feedback reflex pathway (Eilenberg et al., 2010). Here, we set out to conduct a parameter study to specifically examine how NMM reflex Gain and Delay settings effect (i) timing and magnitude of ankle exoskeleton torque and power output and (ii) users’ physiological response – from whole body metabolic rate to individual muscle activity. To do this we implemented an NMM-based controller on a bilateral, tethered robotic ankle exoskeleton and independently varied the reflex Gain (0.8–2.0) and Delay (10–40 ms) while recording the exoskeleton mechanics, and users’ lower-limb joint neuromechanics, muscle activity and whole-body metabolic rate during walking at a fixed 1.25 m/s. In terms of exoskeleton mechanical performance, we hypothesized that increasing NMM reflex Gain at a set Delay would increase both average exoskeleton torque and net power output. On the other hand, we hypothesized that increasing NMM reflex Delay at a set Gain would not affect average exoskeleton torque but still increase net exoskeleton power due to a shift in peak assistance torques toward the period of peak ankle plantarflexion velocities in late stance. In terms of users’ physiological response, we hypothesized that conditions that yielded the most net exoskeleton power would decrease metabolic rate the most (i.e., high Gain = 2.0 and long Delay = 40 ms).

## Materials and Methods

### Powered Ankle Exoskeleton

#### Ankle Exoskeleton Emulator

**FIGURE 1:**
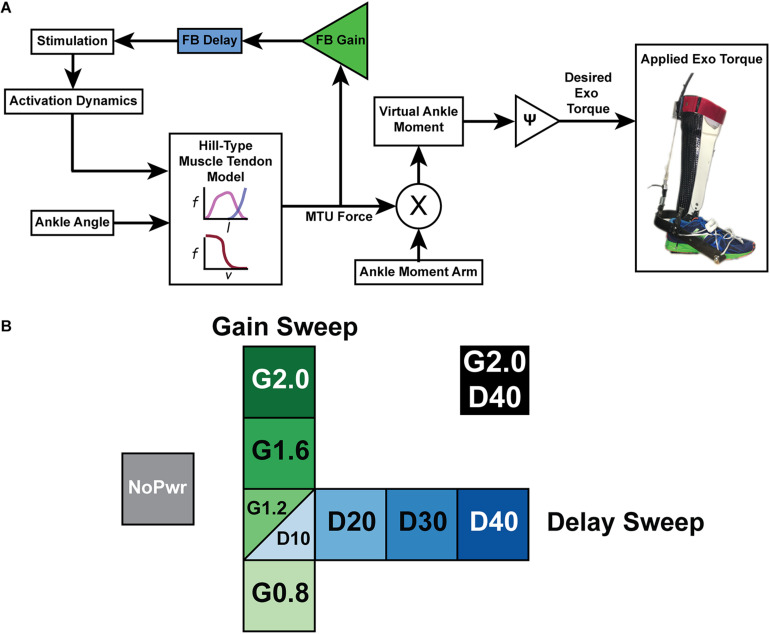
A powered ankle exoskeleton using neuromuscular model (NMM) based control across a range of virtual reflex settings **(A)** Block diagram of a neuromuscular model (NMM) based controller to generate torque output of a powered ankle exoskeleton. The user’s ankle joint angle drives the length change of a virtual muscle-tendon unit (MTU) that uses a positive force feedback reflex loop to stimulate a virtual Hill-type muscle contractile element with force-length and force velocity properties similar to the human plantarflexors. The virtual muscle produces a force that is transmitted through a virtual tendon and then applied through a virtual moment arm to generate a virtual ankle moment which is scaled to produce a desired exoskeleton torque. **(B)** Test conditions for NMM reflex Gain and Delay parameter sweeps included an unpowered or zero-torque condition (NoPwr) (gray) along with powered ankle exoskeleton conditions using controllers with increasing virtual reflex Gain = 0.8, 1.2, 1.6, and 2.0 all with a 10 ms Delay (G0.8, G1.2, G1.6, and G2.0, respectively) (green); increasing virtual reflex Delay = 10, 20, 30, and 40 ms all with a 1.2 reflex Gain (D10, D20, D30, D40, respectively) (blue); and a high-Gain-high-Delay condition (G2.0 D40) (black). All tests were conducted during treadmill walking at 1.25 m/s.

A laboratory-based, tethered exoskeleton emulator provided subjects with plantarflexion torque assistance using a combination of powerful off-board motors (Baldor Electric Co., Fort Smith, AR, United States) and lightweight, bilateral carbon fiber ankle foot orthoses. A flexible Bowden-cable transmission system delivered linear motion from the rotational motion of the motors. The 58” long external conduits (5/16”, Lexco Cable Mfg., Norridge, IL, United States) housed low stretch Vectran rope (V-12 Vectran Single Braid, 3 mm, 1900 lb, West Marine, United States) attached to a moment arm (∼10 cm) at the rear of the exoskeleton (Figure 1A, right). Load cells (500 Hz, LCM Systems Ltd., United Kingdom) were placed in series with the force transmission cables and series elastic element. Goniometers (500 Hz, Biometrics, United Kingdom) were attached to the exoskeleton joint to provide real-time ankle angle information. The control model, designed in Simulink (MathWorks, United States), was embedded on a real-time computer (dSPACE, Germany) that handled analog sensor data sampled at 5 kHz and generated motor commands at 500 Hz. Motor commands were implemented via motor driver (ABB, Cary, NC, United States) operating in velocity control mode.

#### Exoskeleton Neuromuscular Model (NMM) Controller

We implemented a neuromuscular model (NMM) based feedback controller during stance phase with features similar to a previous implementation on a powered ankle-foot prosthesis (Eilenberg et al., 2010; Geyer and Herr, 2010), and recently demonstrated on a tethered ankle exoskeleton (Zhang et al., 2015, 2017a; Witte, 2018; Figure 1A). The emulated plantarflexor muscle tendon unit (MTU) was based off a Hill-type muscle model that consisted of a contractile element (CE), possessing both active and passive properties, and a series elastic element (SEE) modeling the tendon (Zajac, 1989). The internal states of the muscle-tendon model were calculated in a given time-step i (Figure 1B), such that the length of the MTU was a function of modeled musculoskeletal geometry and ankle angle (Eq. 1). SEE length was calculated by subtracting CE length from MTU length (Eq. 2).

(1) $$L_{M\,T\,U,i}=f\,\left(\theta_{a\,n\,k},r_{a\,n\,k}\right)$$

(2) $$L_{S\,E\,E,i}=L_{M\,T\,U,i}-L_{C\,E,i}$$

The force developed in the MTU (*F*_*MTU*_) was a function of the modeled nonlinear stiffness of the SEE and the calculated strain in the SEE (Eq. 3).

(3) $$F_{M\,T\,U,i}=f\,\left(k_{S\,E\,E,i},L_{S\,E\,E,i}\right)$$

Contractile element velocity was calculated from muscle force-length, force-velocity, and activation relationships derived from the Hill model (Eq. 4). The parameters of the lumped-plantarflexors (e.g., *F*_*max*_ = 6000N, *L*_*o*_ = 0.04m, *V*_*max*_ = 0.326 m/s, *k*_*SEE*_ = 315.4 N/mm) were all taken from our previous modeling and simulation work (Sawicki and Khan, 2016). The CE velocity was then integrated to calculate the length of the CE in the next time step (*i+1*) (Eq. 5).

(4) $${\dot{L}}_{C\,E,i}=f\,\left(F-L,\text{F-V},a_{i}\right)$$

(5) $$L_{C\,E,i+1}=\int {\dot{L}}_{C\,E}\,dt$$

In the reflex pathway, *F*_*MTU*_ (Figure 1A) was normalized by a*F_max_*, multiplied by a feedback Gain, and then subjected to a Delay to emulate a positive force feedback neural input signal (Stimulation) (Figure 1A). The feedback loop was closed by modeling the activation dynamics (*a*) of the CE and integrating to find a new activation level at time *i+1* (Eq. 6).

(6) $$a_{i+1}=\int {\dot{a}}_{i}\,dt$$

Finally, the desired exoskeleton torque assistance was set using a gain ψ to set the fraction of the estimated biological torque coming from the NMM (Eq. 7) (Figure 1B).

(7) $$\tau_{e\,x\,o}=F_{M\,T\,U}\times r_{a\,n\,k\,l\,e}\times \psi$$

Based on our own pilot experiments to qualitatively examine user preference/comfort and others’ work indicating that ankle exoskeleton torques that are optimal for reducing metabolic energy cost of walking rarely, if ever, exceed 50% of the biological ankle moment (Koller et al., 2015; Galle et al., 2017; Zhang et al., 2017b), we fixed the value ψ = 0.5 in this study.

During swing phase, we implemented a slack adjustment controller. This slack adjustment controller lengthened the exoskeleton Bowden cable at a constant speed, allowing a larger dorsiflexion range of motion while retaining tension on the cable. We tuned the slack adjustment speed during pilot studies to retain sufficient tension on the cable while minimizing resistance to the user.

### Experimental Protocol

#### Participants

Nine able-bodied participants (age = 24 ± 3 years; mass = 71.3 ± 7.1 kg; height = 1.76 ± 0.05 m; mean ± s.d.) signed a consent form to participate in this study. All consent forms and testing procedures were approved by the University of North Carolina, Chapel Hill and North Carolina State University institutional review board and followed the procedures outlined by the Declaration of Helsinki.

#### Testing Procedure

All trials were completed on an instrumented treadmill (Bertec Inc., Columbus, OH, United States) at a fixed walking speed of 1.25 m/s. First, subjects walked for 25 min with bilateral ankle exoskeletons powered in a baseline condition (Gain = 1.2; Delay = 10 ms) to get comfortable using the device. Baseline values for the parameter sweeps (Gain = 1.2 and Delay = 10 ms) were based on the optimal values of a NMM controller that generated biological plantarflexor torque outputs in previous work using in powered ankle foot prosthesis (Eilenberg et al., 2010; Geyer and Herr, 2010; Markowitz et al., 2011). We chose a 25 min acclimation period based on previous research indicating that subjects need ∼20–30 min of walking to reach steady state metabolic effort in powered ankle exoskeletons (Galle et al., 2013). Subjects were encouraged to experiment with different gaits during this time, but no further instructions were given. Next, each participant walked during nine different experimental conditions lasting 7 min each while wearing the exoskeletons. The nine exoskeleton conditions consisted of one unpowered (NoPwr) where we applied zero-torque; four powered conditions with different NMM reflex Gain (0.8–2.0 = G0.8, G1.2, G1.6 and G2.0) at a set delay (10 ms); four powered conditions with different NMM reflex Delay (10–40 ms = D10, D20, D30, D40) at a set gain (1.2), and a final condition with the highest gain and delay (2.0 gain/40 ms delay = (G2.0/D40) (Figure 1B). The conditions were applied in a pseudo-randomized order. Participants wore a safety harness to decrease the risk of falling or sustaining an injury. The harness did not provide any body weight support. Subjects were instructed to only use the handrails for small balance corrections throughout the trials.

### Measured Outcomes

#### Lower-Limb Joint and Exoskeleton Mechanics

We collected anthropometric data for each subject before testing started. Reflective markers were placed on the left and right anterior superior iliac spine, greater trochanters, medial and lateral epicondyles of the knee, medial and lateral malleoli of the ankle, third metatarsophalangeal joint of the toe, and posterior calcaneus of the heel. Four marker clusters were placed on rigid plates and attached to the pelvis, thighs, shanks, and feet. An eight-camera motion analysis system (Vicon Inc., Oxford, United Kingdom) captured the position of 44 reflective markers at 120 Hz.

To assess lower limb joint kinematics/kinetics, we used a seven-segment rigid body model composed of two thighs, two shanks, two feet, and one pelvis. Raw marker positions were filtered using a second-order low pass Butterworth filter with a cut-off frequency of 10 Hz. Ankle, knee, and hip joint angles were computed as the orientation of the distal segment with reference to the proximal segment. The results reported in this study are of the right leg only. Lower-limb joint moments were computed using standard inverse dynamics analyses (Visual 3D, C-Motion Inc., Germantown, MD, United States). Ankle exoskeleton torque was calculated by multiplying the recorded tension in the exoskeleton Bowden cable (Omegadyne Inc., Norwalk, CT, United States) by the moment arm length to the user’s ankle joint center. Lower-limb joint moments and ankle exoskeleton torque were multiplied by corresponding joint angular velocities to calculate lower-limb joint and exoskeleton mechanical power output. Next, moments/torques/powers were normalized to each subject’s body mass. The biological contribution to total ankle joint moment/power was found by subtracting the measured exoskeleton torque/power from the inverse-dynamics derived, total ankle joint moment/power. Then, stride average, normalized lower-limb joint and exoskeleton moments/torques/powers were obtained by averaging ∼10 representative strides for each subject in each condition. For each subject in each condition, average normalized ankle joint moment/exoskeleton torque was calculated as the integral of the joint moment/exoskeleton torque time-series over the gait cycle divided by the stride time. Net mechanical power outputs at each joint and for the exoskeleton were calculated as the integral of the joint mechanical power time-series over the gait cycle divided by the stride time.

#### Ankle Muscle Activity

Ankle joint muscle activity was measured using surface electromyography (EMG). Wired surface electrodes (SX230, Biometrics Ltd., Newport, United Kingdom), sampled at a frequency of 960 Hz, were placed on the lateral aspect of the soleus (SOL), the medial and lateral gastrocnemius (MG and LG, respectively) and the tibialis anterior (TA) of the right leg. The EMG signals were high-pass filtered with a cutoff frequency 20 Hz, rectified, and low-pass filtered with a cutoff frequency of 6 Hz to get EMG envelopes over the gait cycle. Next, for each muscle, envelopes were normalized to the peak activity observed during walking with zero exoskeleton torque (NoPwr condition). Then, stride average normalized EMG envelopes were obtained by averaging ∼10 representative strides for each subject in each condition. Finally, for each subject in each condition, average normalized EMG activity for each muscle was calculated as the integral of the normalized EMG envelope time-series over the gait cycle divided by the stride time. The EMG data for Subject 2 was determined to be a statistical outlier and was omitted from reported EMG data and associated statistical analyses.

#### Whole-Body Metabolic Rate

Users’ whole-body metabolic rate was estimated using indirect calorimetry. A portable metabolic system (Oxycon Mobile, Viasys Healthcare Inc., Yorba Linda, CA, United States) was used to record the flow rates for oxygen inspired and carbon dioxide expelled. These flow rates were converted to a metabolic rate (Watts) using the Brockway equation (Brockway, 1987) and then normalized to the subject’s body mass (Watts/kg). The metabolic rate from the last 2 min of each 7-min trial were averaged to calculate the steady-state metabolic rate for each condition. The metabolic rate data for Subject 4 was determined to be a statistical outlier and was omitted from reported metabolic rate data and associated statistical analyses.

### Statistical Analyses

Formal comparisons between powered ankle exoskeleton NMM control parameter conditions were made by comparing subject averages for exoskeleton mechanics, ankle joint mechanics, ankle joint muscle activity and metabolic rate across the test conditions (see above for details). Standard error of the mean was used to represent variability between subjects. Two separate, single-factor, repeated-measures ANOVA analyses were performed to test the significance of trends in each measured outcome across NMM controllers; one to test for an effect of NMM reflex Gain (G0.8, G1.2, G1.6, and G2.0) and one to test for an effect of NMM reflex Delay (D10, D20, D30, D40) on exoskeleton mechanics and user’s physiological response. For physiological variables, the NoPwr condition was included for both Gain and Delay tests. Metrics that had a significant main effect (ANOVA; *p* < 0.05) were followed by *post hoc* pairwise comparisons between individual conditions. A Bonferroni correction was applied to account for multiple comparisons. Finally, several *post hoc* least-squares linear regression (LSLR) analyses were performed to quantify the relationship between changes in users’ metabolic rate and changes in users’ neuromechanics with respect to the unpowered exoskeleton condition. *R*^2^ values are reported only when regressions were deemed statistically significant (*p* < 0.05).

## Results

### Ankle Kinematics

**FIGURE 2:**
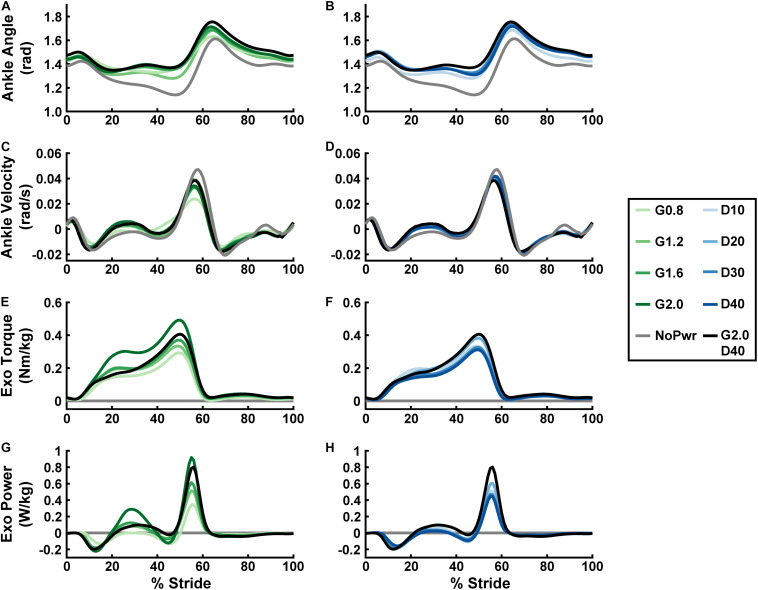
Users’ ankle joint kinematics, and exoskeleton mechanics over a stride cycle. Measurements of users’ ankle angle **(A,B)**, ankle angular velocity **(C,D)**, ankle exoskeleton torque **(E,F)**, and ankle exoskeleton mechanical power **(G,H)** over a stride from heel strike (0%) to heel strike (100%) of the same leg are shown. Ankle plantarflexion is depicted as positive and dorsiflexion as negative for the angle, angular velocity, and torque plots. Positive/negative power indicates net energy transfer from exoskeleton to user and vice versa. All measurements are averages across the study participants (*N* = 9) in each condition with varying neuromuscular model (NMM) controller reflex Gain (green) in left panel **(A,C,E,G)** and Delay (blue) in the right panel **(B,D,F,H)**. Conditions are abbreviated and color coded as follows: unpowered (NoPwr) (gray), NMM reflex Gains of 0.8, 1.2, 1.6, and 2.0 all with reflex Delay = 10 ms (G0.8, G1.2, G1.6, and G2.0, respectively) (green), NMM reflex Delays of 10, 20, 30, and 40 ms all with a reflex Gain = 1.2 (D10, D20, D30, D40, respectively) (blue), and a high-Gain-high-Delay condition (G2.0 D40) (black).

Users assumed a more plantarflexed posture while walking with powered versus unpowered ankle exoskeletons (Figures 2A,B and Supplementary Figure 2). Qualitatively, plantarflexion bias tended to increase when increasing either neuromuscular model (NMM) controller reflex Gain (Figure 2A) or reflex Delay (Figure 2B) and was apparent throughout the entire gait cycle, including times of peak plantarflexion and even during swing phase (Figure 2A). Peak ankle angular velocities (both plantar- and dorsiflexion) decreased from the unpowered to the powered conditions, with increasing Gain (Figure 2C) having a larger effect, qualitatively, than increasing Delay (Figure 2D).

### Exoskeleton Mechanics

**FIGURE 3:**
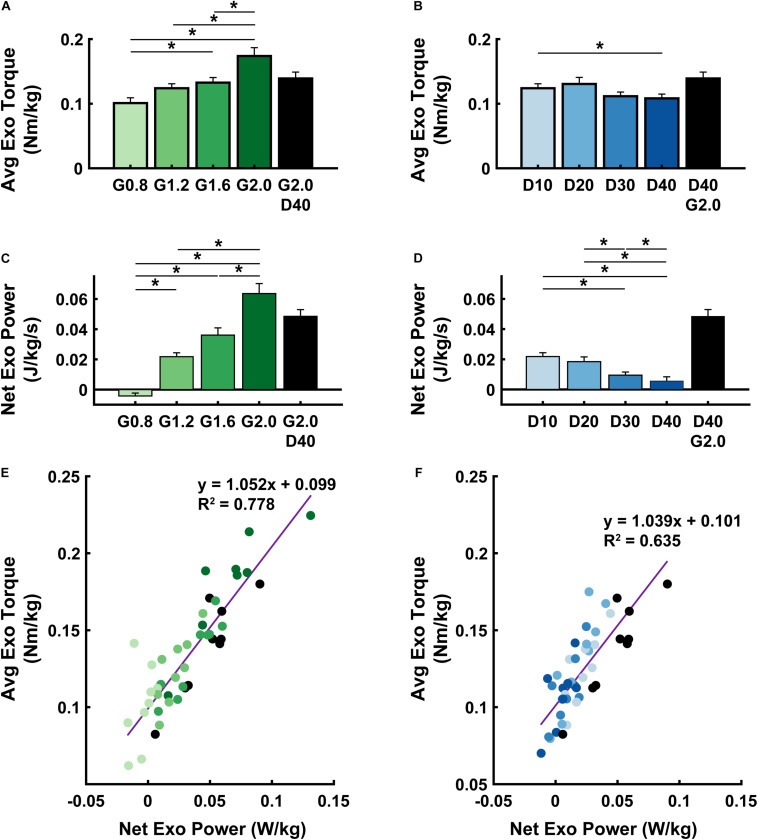
Exoskeleton mechanics. Average (± S.E.M., *N* = 9 participants) exoskeleton torque **(A,B)**, net exoskeleton power **(C,D)**, and a linear regression between the two **(E,F)** across NMM controller reflex Gain (green) **(A,C,E)** and Delay (blue) **(B,D,F)** conditions. * denotes pairwise significant difference with *p* < 0.05. *R*^2^ values are denoted with a “∼” if the linear regression was not statistically significant. Conditions are abbreviated and color coded as follows: NMM reflex Gains of 0.8, 1.2, 1.6, and 2.0 all with reflex Delay = 10 ms (G0.8, G1.2, G1.6, and G2.0, respectively) (green), NMM reflex Delays of 10, 20, 30, and 40 ms all with a reflex Gain = 1.2 (D10, D20, D30, D40, respectively) (blue), and a high-Gain-high-Delay condition (G2.0 D40) (black).

Powered ankle exoskeleton torque and net mechanical power output were both modulated by changes in neuromuscular model (NMM) controller reflex Gain and reflex Delay parameters (Figures 2E–H, 3). Increasing Gain increased both exoskeleton average torque (ANOVA, *p* < 0.001) (Figure 3A) and net power output (ANOVA, *p* < 0.001) (Figure 3C). All Gain conditions produced significantly different average torques except G1.2 compared to G0.8 and G1.6 (paired *t*-test, *p* = 0.187 and *p* = 1.000, respectively) (Figure 3A). Similarly, all net power outputs were significantly different except G1.2 and G1.6 (paired *t*-test, *p* = 0.073). In addition, G0.8 provided net negative power (−0.01 ± 0.00 W/kg) while all others produced net positive power (Figure 3C).

Increasing Delay decreased exoskeleton average torque (ANOVA, *p* = 0.004) (Figure 3B), and net power output (ANOVA, *p* < 0.001) (Figure 3D). Increasing Delay from 10 to 40 ms significantly reduced average exoskeleton torque by ∼12.6% (paired *t*-test, *p* = 0.030) (Figure 3B). Increasing Delay produced significantly different net exoskeleton power between all conditions except D10 to D20 (paired *t*-test, *p* = 1.000) (Figure 3D).

When viewing the interaction between exoskeleton torque and power for both the Gain (Figure 3E) and Delay (Figure 3F) parameter sweeps, there was a near 1:1 positive relationship between net exoskeleton power (W/kg) and average exoskeleton torque (Nm/kg). Each relationship was statistically significant (Gain: LLSR, *p*< 0.0001; *R*^2^ = 0.7784 and Delay: LLSR, *p* < 0.0001; *R*^2^ = 0.6351).

The internal states of the neuromuscular model (NMM) that generated exoskeleton torque output through time across conditions are summarized in Supplementary Figure 1.

### Metabolic Rate

**FIGURE 4:**
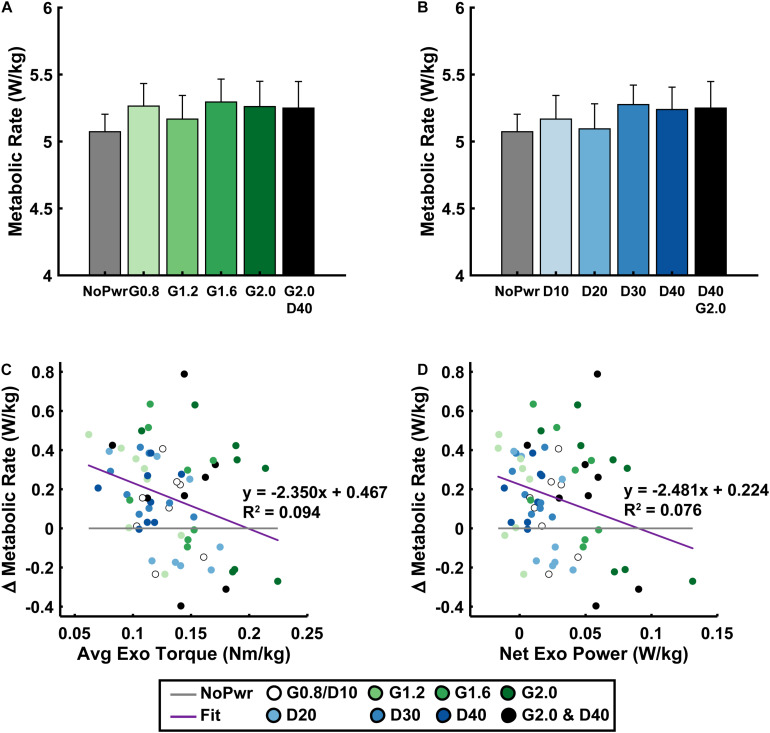
Users’ metabolic rate. Average (± S.E.M., *N* = 9 participants) metabolic rate across NMM reflex Gain (green) **(A)** and Delay (blue) **(B)** conditions. Linear regressions between the change in metabolic rate versus the change in average exoskeleton torque with respect to the unpowered (NoPwr) condition **(C)** and the change in metabolic rate versus the change in net exoskeleton power with respect to the unpowered (NoPwr) condition **(D)**. * denotes pairwise significant difference with *p* < 0.05. *R*^2^ values are denoted with a “∼” if the linear regression was not statistically significant. Conditions are abbreviated and color coded as follows: unpowered (NoPwr) (gray), NMM reflex Gains of 0.8, 1.2, 1.6, and 2.0 all with reflex Delay = 10 ms (G0.8, G1.2, G1.6, and G2.0, respectively) (green), NMM reflex Delays of 10, 20, 30, and 40 ms all with a reflex Gain = 1.2 (D10, D20, D30, D40, respectively) (blue), and a high-Gain-high-Delay condition (G2.0 D40) (black).

Users’ metabolic rate was unchanged when walking with powered ankle exoskeletons using neuromuscular model (NMM) based control across a range of parameter settings (Figure 4). Neither increasing NMM reflex Gain (ANOVA, *p* = 0.1535) (Figure 4A) or reflex Delay (ANOVA, *p* = 0.0558) (Figure 4B) had a significant effect on users’ metabolic rate. Metabolic rate varied slightly across NMM reflex parameter space compared to the NoPwr condition. The D20 condition yielded the lowest average metabolic rate at 5.1 ± 0.2 (a <1.0% increase from NoPwr) and the G1.6 condition yielded the highest metabolic rate at 5.3 ± 0.2 (a 4.4% increase from NoPwr) (Figures 4A,B).

Changes in users’ metabolic rate (i.e., Δ with respect to NoPwr) were not well correlated with standard measures of exoskeleton mechanical assistance across Gain and Delay parameter space. For example, changes in users’ metabolic rate were negatively correlated with both average exoskeleton torque (LLSR, *p* = 0.0136; *y* = −2.350× + 0.467) (Figure 4C), and net exoskeleton power (LLSR, *p* = 0.0275; *y* = −2.482× + 0.224) (Figure 4D), but these significant relationships explained only a small amount of the variability in metabolic rate (*R*^2^ = 0.094 and *R*^2^ = 0.076, for average torque and net power, respectively).

### Biological Ankle Mechanics

**FIGURE 5:**
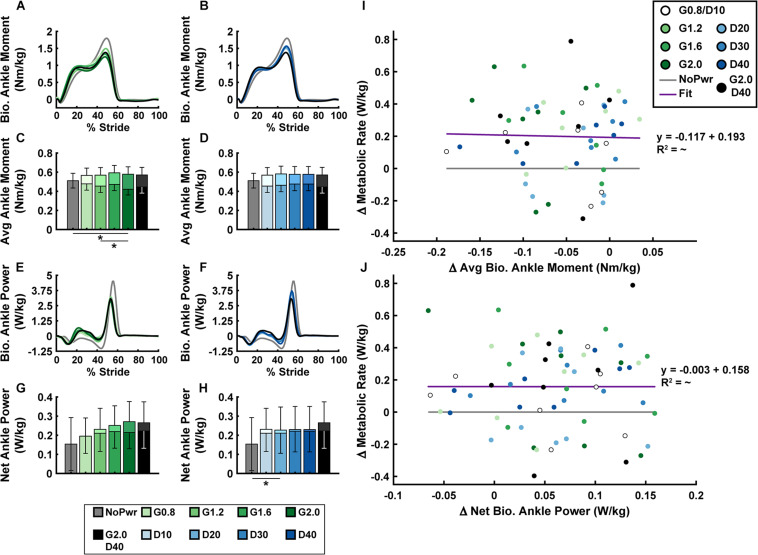
Users’ biological ankle mechanics versus metabolic rate. Measurements of users’ average biological ankle moment **(A,B)** and power **(E,F)** over a stride from heel strike (0%) to heel strike (100%) of the same leg are shown. Ankle plantarflexor torque is depicted as positive. Positive/negative power indicates net energy generation/absorption by the biological structures acting about the ankle. Bar graphs are average biological (darker bars in front) and total = bio + exo (lighter bars in back) ankle moment **(C,D)** and net ankle power **(G,H)**. All measurements are averages across the study participants (*N* = 9) in each condition with varying neuromuscular model (NMM) controller reflex Gain (green) in left panel **(A,C,E,G)** and Delay (blue) in the right panel **(B,D,F,H)**. Linear regressions between the change in metabolic rate versus the change in average biological ankle moment with respect to the unpowered (NoPwr) condition **(I)** and the change in metabolic rate versus the change in net biological ankle power with respect to the unpowered (NoPwr) condition **(J)**. * denotes pairwise significant difference of *p* < 0.05. *R*^2^ value is denoted with a “∼” if the linear regression was not statistically significant. Conditions are abbreviated and color coded as follows: unpowered (NoPwr) (gray), NMM reflex Gains of 0.8, 1.2, 1.6, and 2.0 all with reflex Delay = 10 ms (G0.8, G1.2, G1.6, and G2.0, respectively) (green), NMM reflex Delays of 10, 20, 30, and 40 ms all with a reflex Gain = 1.2 (D10, D20, D30, D40, respectively) (blue), and a high-Gain-high-Delay condition (G2.0 D40) (black).

Users’ biological ankle moment and mechanical power were both modulated by changes in powered ankle exoskeleton NMM controller reflex Gain and Delay parameters (Figure 5 and Supplementary Figure 2). In general, during powered conditions, biological ankle moment (Figures 5A–D) increased during the first half of stance (0–30% stride) and decreased during the second half of stance (30–60% stride), but the timing of peak biological ankle moment was unchanged (Figures 5A,B). Increasing either Gain (ANOVA, *p* < 0.001) (Figure 5C) or Delay (ANOVA, *p* = 0.020) (Figure 5D) caused a decrease in average biological ankle moment. The largest Gain condition (G2.0) was the only powered condition significantly different from the NoPwr condition (paired *t*-test, *p* = 0.005) (Figure 5C), decreasing biological moment by 17.6%. Overall, changes in average biological ankle moment were not significantly correlated with changes in metabolic rate (LLSR, *p* = 0.855) (Figure 5I).

For biological ankle power output (Figures 5E–H), when the exoskeletons were powered on, users exhibited attenuated negative biological ankle power during the early to mid-stance energy storage phase (15–50% stride) and attenuated positive biological ankle power during late-stance push-off phase (50–60% stride). Increasing either the Gain (ANOVA, *p* = 0.009) (Figure 5G) or Delay (ANOVA, *p* = 0.007) (Figure 5H) caused an increase in net biological ankle power. This trend toward net positive biological ankle power was accompanied by a shift in the timing of peak power generation earlier in the stride during powered conditions (Figures 5E,F). Similar to average biological ankle moment, changes in net biological ankle power were not significantly correlated with changes in metabolic rate (LLSR, *p* = 0.996) (Figure 5J).

Knee and hip joint mechanics are summarized in Supplementary Figures 3, 4. Qualitatively, with exoskeletons powered on, users assumed a more extended knee posture throughout the walking stride and exhibited larger knee flexion moments during stance compared to the unpowered condition. The knee joint absorbed more energy between 15 and 40% of the stride and generated more energy between 40 and 60% of the stride. At the hip, users assumed a more extended posture at peak flexion and extension. Although not statistically analyzed, there was an apparent trend during powered conditions of higher extension moments generated at the hip during early and mid-stance (∼5–40% stride) causing an increase in hip positive power, especially with Delay conditions. Mechanical power, generated at the hip near the end of swing to pre-emptively extend the limb before heel strike (85–100% stride), was amplified in powered conditions. These trends were not examined statistically.

### Ankle Muscle Activity

**FIGURE 6:**
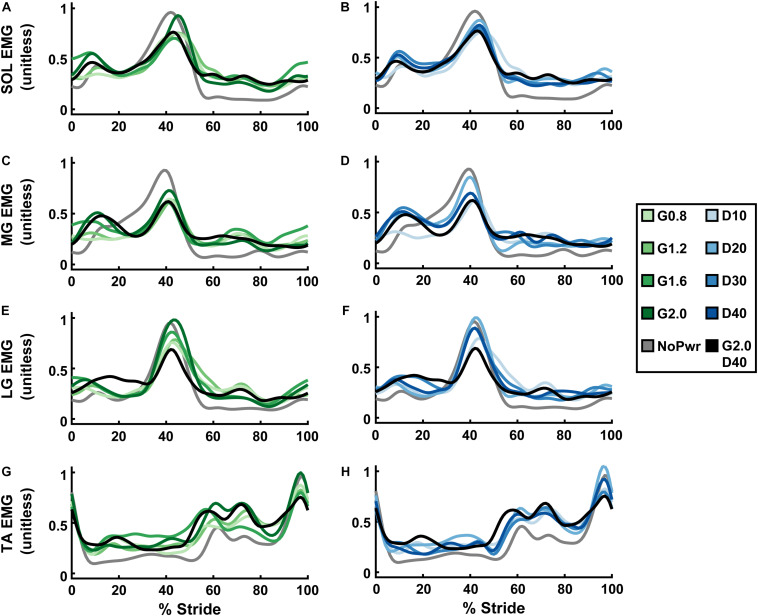
Users’ ankle muscle activity over a stride cycle. Measurements of users’ normalized electromyography (EMG) signals recorded from soleus (SOL; **A,B**), medial gastrocnemius (MG; **C,D**), lateral gastrocnemius (LG; **E,F**), and tibialis anterior (TA; **G,H**) over a stride from heel strike (0%) to heel strike (100%) of the same leg are shown. All measurements are averages across the study participants (*N* = 9) in each condition with varying neuromuscular model (NMM) controller reflex Gain (green) in left panel **(A,C,E,G)** and Delay (blue) in the right panel **(B,D,F,H)**. Conditions are abbreviated and color coded as follows: unpowered (NoPwr) (gray), NMM reflex Gains of 0.8, 1.2, 1.6, and 2.0 all with reflex Delay = 10 ms (G0.8, G1.2, G1.6, and G2.0, respectively) (green), NMM reflex Delays of 10, 20, 30, and 40 ms all with a reflex Gain = 1.2 (D10, D20, D30, D40, respectively) (blue), and a high-Gain-high-Delay condition (G2.0 D40) (black).

**FIGURE 7:**
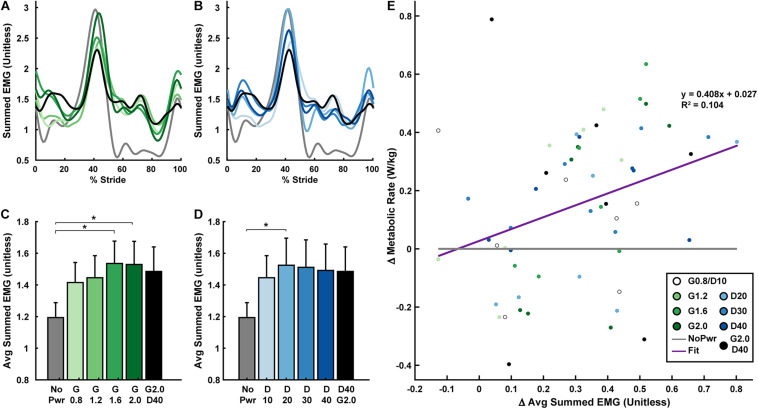
User’s summed ankle muscle activity versus metabolic rate. Measurements of users’ summed normalized electromyography (EMG) signals recorded from soleus + medial gastrocnemius + lateral gastrocnemius + tibialis anterior (SOL+MG+LG+TA) over a stride from heel strike (0%) to heel strike (100%) of the same leg are shown **(A,B)**. Bar graphs are averages of the summed EMG signals over the stride **(C,D)**. All measurements are averages across the study participants (*N* = 9) in each condition with varying neuromuscular model (NMM) controller reflex Gain (green) in left panel **(A,C)** and Delay (blue) in the right panel **(B,D)**. Linear regression between the change in metabolic rate versus the change in average summed EMG with respect to the unpowered (NoPwr) condition **(E)**. * denotes pairwise significant difference of *p* < 0.05. *R*^2^ value is denoted with a “∼” if the linear regression was not statistically significant. Conditions are abbreviated and color coded as follows: unpowered (NoPwr) (gray), NMM reflex Gains of 0.8, 1.2, 1.6, and 2.0 all with reflex Delay = 10 ms (G0.8, G1.2, G1.6, and G2.0, respectively) (green), NMM reflex Delays of 10, 20, 30, and 40 ms all with a reflex Gain = 1.2 (D10, D20, D30, D40, respectively) (blue), and a high-Gain-high-Delay condition (G2.0 D40) (black).

Powered ankle exoskeletons substantially altered users’ ankle muscle activity and qualitative trends were similar across NMM controller Gain and Delay parameters (Figure 6 and Supplementary Figures 5–8). In general, with exoskeletons powered-on, the plantarflexors [i.e., soleus (SOL), medial gastrocnemius (MG), and lateral gastrocnemius (LG)] showed reduced activity during mid- to late stance phase (15–60% stride) and markedly increased activity during swing phase through early stance (60–15% stride) (Figures 6A–F). Summed ankle muscle activity (Figures 7A,B) increased during early stance (0–30% stride) and swing (60–100% stride) with powered assistance. However, during late stance/push-off, summed EMG decreased compared to the unpowered condition. On average (Figures 7C,D), summed ankle muscle activity increased across all powered condition from unpowered. Specifically, G1.6, G20, and D20 significantly increased summed EMG compared to the unpowered condition (paired *t*-test; *p* = 0.003, 0.007, and 0.043, respectively). Average summed ankle EMG was significantly correlated with changes in metabolic cost (LLSR, *p* < 0.001) (Figure 7E).

When viewing average muscle activity of plantarflexor muscles individually; peak SOL (Figures 6A,B and Supplementary Figure 5) and MG activity (Figures 6C,D and Supplementary Figure 6) decreased and occurred later in the gait cycle with powered conditions; peak LG activity (Figures 6E,F and Supplementary Figure 7) increased with increasing Gain. Average LG activity was significantly correlated with metabolic cost (LLSR, *p* < 0.001) (Supplementary Figure 7E).

Dorsiflexor [e.g., tibialis anterior (TA)] activity increased throughout the stride in powered conditions, except at terminal swing (90–100% stride) where it was reduced (Figures 6G,H). On average, TA EMG increased with Gain (ANOVA, *p* < 0.001) compared to NoPwr. Substantial increases in TA activity are seen during early swing phase (60–80% stride). TA EMG was not significantly correlated with metabolic cost (LLSR, *p* = 0.258) (Supplementary Figure 8E).

## Discussion

The purpose of this study was to apply a neuromuscular model (NMM) of the human plantarflexors to control torque output of a powered ankle exoskeleton during walking. As a first step, we developed an NMM comprised of a Hill-type musculotendon driven by a simple positive force feedback reflex loop and examined the effects of the NMM reflex Gain and Delay settings on (i) ankle exoskeleton mechanical performance and (ii) users’ physiological response. First, we hypothesized increasing Gain would consequently increase average exoskeleton torque and net power output while increasing Delay would have no effect on torque but increase net exoskeleton power. Indeed, increasing Gain increased both average torque and net power outputs (Figures 3A,C). Surprisingly, increasing Delay decreased both average torque and net power (Figures 3B,D). We assumed that powered exoskeleton assistance would not affect ankle kinematics when predicting the effects of each control parameter on exoskeleton torque and power. Contrary to our expectation, users assumed a more plantarflexed posture and decreased peak angular velocities when walking with powered assistance (Figures 2A,B). These kinematic changes altered virtual muscle dynamics in the NMM, creating a negative feedback loop with the controller’s torque output. Early onset exoskeleton torque caused a more plantarflexed posture that resulted in shorter active virtual muscle lengths and higher active virtual muscle velocities, which both decreased virtual muscle force output and ongoing virtual muscle reflex activation (i.e., a negative feedback loop) (Supplementary Figure 1). Apparently, there is a more complex relationship between the NMM-based exoskeleton control and the user than we initially expected. Second, we hypothesized conditions with higher net exoskeleton power would result in higher metabolic benefit. Even though we successfully modulated net exoskeleton power output, we found no NMM controller parameter set that reduced metabolic rate (Figures 4A,B).

Maximizing metabolic benefit is not as simple as increasing exoskeleton torque and power delivery. Tuning the timing of assistance is essential. In our study, increasing exoskeleton torque and power output was weakly correlated with reductions in metabolic rate (Figures 4C,D), lending some support for the idea that more positive net exoskeleton power yields higher metabolic benefits (Mooney et al., 2014; Jackson and Collins, 2019). Extrapolating this trend suggests: if our device had generated higher net power, we may have achieved a significant metabolic benefit. Our device achieved average torque magnitude per leg (0.1–0.2 Nm/kg) consistent with Jackson and Collins (2019), resulted in ∼15% reduction with respect to zero torque, ruling out hardware limitations as a source of poor performance. In contrast, torque onset in our study was much earlier (∼10% of stride versus ∼40%) and generated negative power in early stance that acted to counter the impulsive positive power delivered late in stance (Figures 2E–H). Overall, the data suggest that our relatively small net power outputs did not result from lack of torque magnitudes but poor timing. Indeed, there are infinite ways to deliver a given amount of net positive power over a stride. While the difference between pos/neg power is the driving factor, evidence is mounting that the timing of power delivery may be even more critical.

Previous studies have directly examined the relationship between timing of ankle exoskeleton assistance and metabolic cost (Malcolm et al., 2013; Galle et al., 2017) and found that assistance torque onsets between 35 and 45% of the gait cycle are most beneficial. Similarly, using human-in-the-loop optimization to tune timing-based ankle torque assistance on an individual basis, Zhang et al. (2017b) reported the highest metabolic benefits to date (∼24% below unpowered), with torque onsets between 20 and 40% of the walking stride. Furthermore, examining the exoskeleton power vs. time patterns from these aforementioned studies reveals peak positive power delivery to the user’s ankle occurred between 50 and 60% of the gait cycle (i.e., in the “push-off” stage) (Collins et al., 2015; Jackson and Collins, 2015; Galle et al., 2017). Our device also delivered peak positive power focused near push-off (Figures 2G,H). However, our torque onset was much too early, with plantarflexion assistance beginning at ∼5–10% of the gait cycle for all powered conditions (Figures 2E,F). This poor timing resulted in a jerky, oscillatory power delivery as evidenced by a rapid sequence of energy absorption and return to/from the exoskeleton in early stance (Figures 2G,H). Concurrently, a lack of normal ankle dorsiflexion resulting from “too early” exoskeleton plantarflexor torque may have disrupted the normal storage and return of energy in the Achilles tendon. One possibility, while our device hardware was powerful enough, the NMM control scheme yielded “too-much” torque “too-early,” even when the Gain and Delay were set to maximize net power delivery (i.e., G2.0 D40) and this may explain our lack of metabolic benefits. However, a recent study using heuristic coadaptive control (Jackson and Collins, 2019) demonstrated metabolic benefits similar to the human in the loop optimized pattern with bimodal peaks similar to ours. That study highlights the fact that the relationship between user response is complicated, depend on other physiological factors like muscle activity and joint posture.

Analyses that focus only on exoskeleton mechanical performance when evaluating physiological response of the human user may be short sighted. Ultimately, a user’s metabolic cost depends on how a device influences underlying metabolic demand on muscles spanning the lower-limb joints (Beck et al., 2019). Along these lines, perhaps examining changes in users’ limb-joint mechanics (Figure 5) would better reflect changes in metabolic demand than exoskeleton mechanics alone (Figures 3, 4). Numerous studies, focusing on a single locomotion task (e.g., walking on level ground at fixed speed) have shown that the metabolic benefit from exoskeleton assistance is proportional to reductions in biological power output of the target joint (Sawicki and Ferris, 2008; Mooney et al., 2014; Seo et al., 2016). Surprisingly, when we examined study-wide powered exoskeletons conditions (i.e., all NMM controller settings), we did not find significant correlations between changes in users’ metabolic rate and changes in *either* average biological ankle moment (Figure 5I) *or* net power (Figure 5J). It is important to note, that our formal statistical analyses focused on changes in user’s ankle kinetics, but changes in kinetics at proximal joints could have also impacted metabolic demand (Mooney and Herr, 2016). For example, a qualitative glance at knee (Supplementary Figure 3) and hip (Supplementary Figure 4) moments and powers reveals changes in early-mid stance knee kinetics in response to NMM-based ankle assistance. It is important to note, that changes in limb-joint mechanical demand driven by exoskeleton-assistance need not be a good predictor for changes in metabolic demand of the user. This is especially true for assistance applied at the ankle, where limb-joint power may be a poor indicator of muscle contractile dynamics due to presence of the highly elastic Achilles tendon in series (Nuckols and Sawicki, 2020). Focusing directly on muscles may be a more tractable way to gain insight into how exoskeletons alter metabolic demand (Beck et al., 2019).

Muscle activity measurements may be the best way to estimate how exoskeleton assistance impacts muscle-level mechanical and metabolic demand during walking. Various studies have found positive correlations between measures of muscle activity and metabolic rate (Collins et al., 2015; Jackson and Collins, 2015; Zhang et al., 2015; Nuckols and Sawicki, 2020; Nuckols et al., 2020). In this study, we calculated peak summed electromyography (EMG) for the major muscles spanning the ankle joint: soleus (SOL), medial & lateral gastrocnemius (MG & LG), and tibialis anterior (TA). Stride averaged summed EMG increased for all powered conditions, especially during swing phase and into early stance (Figures 7A,B). We found significant least-squares linear regression between changes in summed EMG and changes in metabolic rate due to exoskeleton assistance (Figure 7E). Though summed EMG could only explain 10.4% of the variability in metabolic response to the exoskeletons, this was still higher than the variability explained by either the exoskeleton (Figures 4C,D) or biological ankle joint mechanics (Figures 5I,J). Interestingly, when this same analysis was done for each muscle individually, changes in LG and SOL muscle activity (Supplementary Figures 4, 5), had higher correlation with changes in metabolic rate (*R*^2^ = 0.393 and 0.208, respectively) than the summed activity across *all* of the muscles (*R*^2^ = 0.104). According to Beck et al. (2019), scaling each muscle’s activity by its physiological volume [i.e., physiological cross sectional area (PSCA) × rest length] may yield better estimates of metabolic rate because larger muscles would consume more energy per activation than smaller muscles. Indeed, when we performed a *post hoc* analysis that scaled EMG by relative muscle volumes, and then calculated the correlation between changes in summed muscle activity and changes in metabolic rate our *R*^2^ improved from 0.104 to 0.133. These correlations are still low compared to other reported values [*e.g., R*^2^ ∼0.4 in Sawicki and Ferris (2008), Collins et al. (2015), Jackson and Collins (2015), and Nuckols and Sawicki (2020)]. By incorporating muscles beyond those than span the ankle joint, and including muscle volume scaling, we would expect correlations to continue to improve. Finally, it is important to acknowledge that other studies have stated the duration of the contraction, i.e., accounting for the *rate* of muscle activation (Kram and Taylor, 1990; Griffin et al., 2003), may also be a key factor that can help relate EMG and metabolic cost of muscle contraction. Recently, Nuckols et al. (Nuckols and Sawicki, 2020), employed this technique when analyzing the effects of ankle exoskeleton assistance across speeds and reported *R*^2^ up to 0.69. While these results seem promising, there is still considerable debate on whether cycle averaged (Beck et al., 2019) or step duration averaged (Nuckols and Sawicki, 2020) more accurately depicts muscular energy consumption. Nevertheless, our study and many others are building strong support for using users’ muscle activity rather limb-joint mechanics to explain changes in metabolic cost.

Habituation, or the ability of user to adapt a motor coordination strategy that leverages robotic assistance, is another factor that influences whether an exoskeleton control strategy yields metabolic benefit. A hallmark observation during motor adaptation to exoskeleton assistance is an initial onset of high levels of muscle co-activation both local to the exoskeleton assistance [e.g., tibialis anterior (TA)] and also more globally across the limb (e.g., biceps femoris). Over time, users typically exhibit attenuated co-activation and thus avoid the metabolic penalty associated with the additional muscle activity of antagonist muscles (Cain et al., 2007; Sawicki and Ferris, 2008; Galle et al., 2013; Gordon et al., 2013; Koller et al., 2015; Jackson and Collins, 2019). In this study, across powered conditions, we observed substantial co-activation of plantar- and dorsiflexor muscles over the majority of the stride (Figure 6). Even after 25 min of training in the device, we observed increased TA (dorsiflexor) activation over ∼90% of the stride duration, including during stance (Figures 6G,H and Supplementary Figure 8). In addition, we observed heightened activity of MG, LG, and SOL (plantarflexor) during swing phase (60–100% of stride) (Figures 6A–F and Supplementary Figures 5–7). Two possibilities for persistent co-activation are: we did not give users a long enough time to learn to walk with the NMM controller or NMM-based control is impossible to learn at all.

Though we did not formally examine the time-course of habituation to our device, it is possible that users needed more time to reach the full metabolic benefit of NMM-based control. It is well known that it takes users a significant amount of training time to learn to use robotic exoskeletons. For ankle exoskeletons, the time to reach a walking pattern with new steady state neuromechanics and energetics is on the order of 15 min to 1.5 h, depending on the observed state variable (Cain et al., 2007; Sawicki and Ferris, 2008; Galle et al., 2013; Gordon et al., 2013; Koller et al., 2015, 2017; Nuckols and Sawicki, 2020). In this study we gave users 25 min of training time based on benchmarks indicating that >20 min of exposure is sufficient to ensure full metabolic benefit in an ankle exoskeleton (Galle et al., 2013). However, even after training, we found no NMM control parameters that could significantly reduce users’ metabolic rate (Figure 4). One possibility for poor user performance is that NMM-based control is harder to learn than time-based (Galle et al., 2013) or EMG-driven (Sawicki and Ferris, 2008; Gordon et al., 2013; Koller et al., 2015) controllers. Indeed, there is some evidence that adaptation rate may be specific to the exoskeleton control architecture. For example, data comparing adaptation rate between myoelectric and bang-bang foot-switch control strategies on an ankle exoskeleton indicate that although users reached steady state human-exoskeleton behavior within 30 min with both controllers, it occurred 15 min faster with the bang-bang control (Cain et al., 2007). Even amongst adaptive myoelectric control schemes, heuristics-based control (Jackson and Collins, 2019) converged faster than traditional strategies (Koller et al., 2015). Thus, it is possible that despite its strong grounding in human sensorimotor physiology, our NMM-based controller is less intuitive, making it harder for users to find an efficient movement strategy. Follow up studies could examine the extent to which people can learn NMM-control if given more time and a larger parameter space, perhaps with some guidance to proactively encourage users to broadly explore motor coordination strategies (Selinger et al., 2015, 2019; Wong et al., 2019). It is important to note, it is entirely possible that the structure of NMM-based control is impossible to learn at all. Perhaps, the NMM-control architecture is identified by the human nervous system as a persistent source of uncertainty (i.e., an unidentifiable disturbance), and thus stiffening the joints via co-activation of antagonist muscles is the optimal feed-forward strategy to deal with consequences of inherent sensorimotor delays in the nervous system that would otherwise undermine stable movement (Hogan, 1989), a strategy that may also be the most economical manner to deal with uncertainty.

Based on our results it is tempting to dismiss NMM-based ankle exoskeleton control as an effective strategy for minimizing users’ metabolic cost of walking. However, it is important to note that our study only examines a small subset of possible NMM control architectures in a limited set of locomotion tasks (i.e., fixed speed at 1.25 m/s). For example, in the reflex-based NMM control architecture, torque assistance is predominantly dependent on ankle kinematics which are only indirectly influenced by the user, creating a complex human-machine interaction. More direct control could simplify the human-machine interaction allowing for quicker adaptation to the device and improved metabolic benefit. Along these lines, researchers have recently begun to explore hybrid versions of NMM-based control that directly feed the user’s muscle activity to drive the activation of the muscle-tendon model instead of implementing a positive force feedback reflex pathway (Kirby, 2018). In this case, preliminary results using human in the loop optimization (HiLO) to tune parameters in the controller’s virtual MTU model yield up to 10% reduction in metabolic rate for medium and fast walking speeds. Notably, in that study NMM-based control was still outperformed by pure EMG and time-based controllers implemented on the same subjects and hardware. These results highlight the possibility that the torques that are generated by the NMM architecture may be constrained in such a way that prevents the exoskeleton from delivering the energetically optimal time-based torque profile (Zhang et al., 2017b). Indeed, despite the intuition that applying the physiological torque pattern observed in human gait (*a la* NMM) should provide a sound template for exoskeleton assistance, evidence is growing that physiologically based controllers are not optimal for reducing users’ metabolic cost in unchanging walking environments (e.g., fixed speeds on level ground with even terrain), at least at the ankle joint.

Exoskeletons have utility beyond the context of reducing energy cost of locomotion. Locomotion in natural environments is dynamic and unsteady. A key point often overlooked is that time-based, human-in-the-loop optimized controllers are successful at reducing metabolic cost in steady, unchanging tasks because they provide identical assistance for each step. This provides a consistent platform for quick-and-easy user adaptation to an efficient motor strategy, but inherently lacks the versatility to adapt to even slight changes in either the user or the environment. In natural locomotion, gait speed, ground slope, and smoothness of the terrain change from step to step and control strategies must progress to be versatile in dynamic environments. Despite its potential drawbacks in steady conditions, NMM-based control may be ideal for more dynamic tasks. For example, NMM-based control on robotic prostheses can robustly restore lost function in people with amputation by automatically adapting assistance over changing speeds without explicit changes in the control parameters (Eilenberg et al., 2010; Markowitz et al., 2011). It is possible that NMM-based control of exoskeletons is equally adaptive, and future studies are needed to examine the extent to which NMM controllers can respond directly to rapid changes in a user’s state (e.g., kinematics or muscle activity). In short, exploring a broader lens of applications that include a diverse, dynamically changing locomotion task-environment space will truly define the capability of NMM-based exoskeleton control to augment movement outside of the laboratory.

## Conclusion

Our novel neuromuscular model (NMM)-based ankle exoskeleton controller can provide a wide range of assistance torque and power through changes in the virtual reflex feedback Gain and Delay. While these changes did not elicit metabolic benefits, our analysis provides further insight into the complex interactions between a human user and their exoskeleton. Although users were able to adapt their lower-limb motor behaviors during powered conditions, the new strategy they settled to did not confer a metabolic benefit. According to our analysis of muscle activity patterns, co-activation of ankle plantar-dorsiflexor muscles persisted even after significant training time, indicating that adaptation to the NMM control architecture was not as intuitive as we expected given its direct analogy to the underlying physiology. NMM-based exoskeleton control may still be useful in contexts where torque assistance must be automatically and continuously responsive to rapid changes in the state of environment or user.

## Data Availability Statement

The original contributions presented in the study are included in the article/Supplementary Material, further inquiries can be directed to the corresponding author.

## Ethics Statement

The studies involving human participants were reviewed and approved by the University of North Carolina, Chapel Hill and North Carolina State University Institutional Review Board. The patients/participants provided their written informed consent to participate in this study.

## Author Contributions

BS contributed to the data and statistical analyses, data interpretation, and editing of the manuscript and figures. SP led the data collection, performed initial data analysis, and drafted an early version of the manuscript. RN implemented the exoskeleton controller and assisted with data collection and analysis. JM assisted with the data collection and analysis. AY contributed to the statistical analysis and data interpretation and edited the manuscript. GS led the conceptual design of the study, provided oversight during controller development and data collection, contributed to data analysis, interpretation and presentation and edited the manuscript. All authors read and approved the final version of the manuscript.

## Conflict of Interest

The authors declare that the research was conducted in the absence of any commercial or financial relationships that could be construed as a potential conflict of interest.
