# Supplementary material for: Neuromechanics and Energetics of Walking With an Ankle Exoskeleton Using Neuromuscular-Model Based Control: A Parameter Study
Source: Front Bioeng Biotechnol. 2021 Apr 9;9:615358. doi: 10.3389/fbioe.2021.615358 (PMC8091965; doi:10.3389/fbioe.2021.615358)
Supplement: Supplementary file 2 [file Data_Sheet_2.zip › Supplementary Figure Captions.DOCX]

**Supplementary Figure 1. Neuromuscular model (NMM) muscle-tendon (MT) dynamics over a stride cycle.** Records of the input and output signals to and from the ankle exoskeleton neuromuscular model controller (Fig. 1). The neural feedback (FB) signal for activating the Hill-type muscle (A and B), the output force generated by the virtual muscle-tendon unit (MTU) (C and D), the length (L_CE_) [with right axis normalized to the muscle optimal length] (E and F) and velocity (V_CE_) [with right axis normalized to the maximum muscle velocity (shortening is negative)] (G and H) of the virtual muscle contractile element (CE) and the mechanical power generated by the virtual MTU (I and J). All data were first averaged across many strides per participant and then across all participants (N=9) per condition. Records across Gain conditions can be seen on the left (A, C, E, G, I) and across Delay conditions on the right (B, D, F, H, J). Conditions are abbreviated and color coded as follows: NMM reflex Gains of 0.8, 1.2, 1.6, and 2.0 all with reflex Delay = 10ms (G0.8, G1.2, G1.6, and G2.0, respectively) (green), NMM reflex Delays of 10, 20, 30, and 40ms all with a reflex Gain = 1.2 (D10, D20, D30, D40, respectively) (blue), and a high-Gain-high-Delay condition (G2.0 D40) (black).

**Supplementary Figure 2. Users’ total (bio + exo) ankle kinematics and kinetics over a stride cycle.** Measurements of users’ ankle angle (A and B), total ankle moment (C and D), and total ankle power (E and F) over a stride from heel strike (0%) to heel strike (100%) of the same leg are shown. Ankle plantarflexion is depicted as positive and dorsiflexion as negative for the angle and moment plots. All data were first averaged across many strides per participant and then across all participants (N=9) per condition. Time-series data across Gain conditions can be seen on the left (A, C, and E) and across Delay conditions on the right (B, D, and F). Conditions are abbreviated and color coded as follows: unpowered (NoPwr)(gray), NMM reflex Gains of 0.8, 1.2, 1.6, and 2.0 all with reflex Delay=10ms (G0.8, G1.2, G1.6, and G2.0, respectively) (green), NMM reflex Delays of 10, 20, 30, and 40ms all with a reflex Gain=1.2 (D10, D20, D30, D40, respectively) (blue), and a high-Gain-high-Delay condition (G2.0 D40) (black).

**Supplementary Figure 3. Users’ knee kinematics and kinetics over a stride cycle.** Measurements of users’ knee angle (A and B), knee moment (C and D), and knee power (E and F) over a stride from heel strike (0%) to heel strike (100%) of the same leg are shown. Knee flexion is depicted as positive and extension as negative for the angle and moment plots. All data were first averaged across many strides per participant and then across all participants (N=9) per condition. Time-series data across Gain conditions can be seen on the left (A, C, and E) and across Delay conditions on the right (B, D, and F). Conditions are abbreviated and color coded as follows: unpowered (NoPwr)(gray), NMM reflex Gains of 0.8, 1.2, 1.6, and 2.0 all with reflex Delay=10 ms (G0.8, G1.2, G1.6, and G2.0, respectively) (green), NMM reflex Delays of 10, 20, 30, and 40 ms all with a reflex Gain=1.2 (D10, D20, D30, D40, respectively) (blue), and a high-Gain-high-Delay condition (G2.0 D40) (black).

**Supplementary Figure 4. Users’ hip kinematics and kinetics over a stride cycle.** **.** Measurements of users’ hip angle (A and B), hip moment (C and D), and hip power (E and F) over a stride from heel strike (0%) to heel strike (100%) of the same leg are shown. Hip flexion is depicted as negative and extension as positive for the angle and moment plots. All data were first averaged across many strides per participant and then across all participants (N=9) per condition. Time-series data across Gain conditions can be seen on the left (A, C, and E) and across Delay conditions on the right (B, D, and F). Conditions are abbreviated and color coded as follows: unpowered (NoPwr) (gray), NMM reflex Gains of 0.8, 1.2, 1.6, and 2.0 all with reflex Delay=10 ms (G0.8, G1.2, G1.6, and G2.0, respectively) (green), NMM reflex Delays of 10, 20, 30, and 40 ms all with a reflex Gain=1.2 (D10, D20, D30, D40, respectively) (blue), and a high-Gain-high-Delay condition (G2.0 D40) (black).

**Supplementary Figure 5. Users’ soleus muscle activity.** Measurements of users’ normalized soleus (SOL) electromyography (EMG) over a stride from heel strike (0%) to heel strike (100%) of the same leg are shown (A, B). Bar graphs are averages of the normalized SOL EMG signal over the stride (C and D). All measurements are averages across the study participants (N=9) in each condition with varying neuromuscular model (NMM) controller reflex Gain (green) in left panel (A, C) and Delay (blue) in the right panel (B, D). Linear regression between the change in metabolic rate versus the change in average SOL EMG with respect to the unpowered (NoPwr) condition (E). * denotes pairwise significant difference of p < .05. R^2^ value is denoted with a “~” if the linear regression was not statistically significant. Conditions are abbreviated and color coded as follows: unpowered (NoPwr) (gray), NMM reflex Gains of 0.8, 1.2, 1.6, and 2.0 all with reflex Delay=10 ms (G0.8, G1.2, G1.6, and G2.0, respectively) (green), NMM reflex Delays of 10, 20, 30, and 40 ms all with a reflex Gain=1.2 (D10, D20, D30, D40, respectively) (blue), and a high-Gain-high-Delay condition (G2.0 D40) (black).

**Supplementary Figure 6. Users’ medial gastrocnemius muscle activity.** Measurements of users’ normalized medial gastrocnemius (MG) electromyography (EMG) over a stride from heel strike (0%) to heel strike (100%) of the same leg are shown (A, B). Bar graphs are averages of the normalized MG EMG signal over the stride (C and D). All measurements are averages across the study participants (N=9) in each condition with varying neuromuscular model (NMM) controller reflex Gain (green) in left panel (A, C) and Delay (blue) in the right panel (B, D). Linear regression between the change in metabolic rate versus the change in average SOL EMG with respect to the unpowered (NoPwr) condition (E). * denotes pairwise significant difference of p < .05. R^2^ value is denoted with a “~” if the linear regression was not statistically significant. Conditions are abbreviated and color coded as follows: unpowered (NoPwr) (gray), NMM reflex Gains of 0.8, 1.2, 1.6, and 2.0 all with reflex Delay=10 ms (G0.8, G1.2, G1.6, and G2.0, respectively) (green), NMM reflex Delays of 10, 20, 30, and 40 ms all with a reflex Gain=1.2 (D10, D20, D30, D40, respectively) (blue), and a high-Gain-high-Delay condition (G2.0 D40) (black).

**Supplementary Figure 7. Users’ lateral gastrocnemius muscle activity.** Measurements of users’ normalized lateral gastrocnemius (LG) electromyography (EMG) over a stride from heel strike (0%) to heel strike (100%) of the same leg are shown (A, B). Bar graphs are averages of the normalized LG EMG signal over the stride (C and D). All measurements are averages across the study participants (N=9) in each condition with varying neuromuscular model (NMM) controller reflex Gain (green) in left panel (A, C) and Delay (blue) in the right panel (B, D). Linear regression between the change in metabolic rate versus the change in average LG EMG with respect to the unpowered (NoPwr) condition (E). * denotes pairwise significant difference of p < .05. R^2^ value is denoted with a “~” if the linear regression was not statistically significant. Conditions are abbreviated and color coded as follows: unpowered (NoPwr) (gray), NMM reflex Gains of 0.8, 1.2, 1.6, and 2.0 all with reflex Delay=10ms (G0.8, G1.2, G1.6, and G2.0, respectively) (green), NMM reflex Delays of 10, 20, 30, and 40ms all with a reflex Gain=1.2 (D10, D20, D30, D40, respectively) (blue), and a high-Gain-high-Delay condition (G2.0 D40) (black).

**Supplementary Figure 8. Users’ tibialis anterior muscle activity.** Measurements of users’ normalized tibialis anterior (TA) electromyography (EMG) over a stride from heel strike (0%) to heel strike (100%) of the same leg are shown (A, B). Bar graphs are averages of the normalized TA EMG signal over the stride (C and D). All measurements are averages across the study participants (N=9) in each condition with varying neuromuscular model (NMM) controller reflex Gain (green) in left panel (A, C) and Delay (blue) in the right panel (B, D). Linear regression between the change in metabolic rate versus the change in average TA EMG with respect to the unpowered (NoPwr) condition (E). * denotes pairwise significant difference of p < .05. R^2^ value is denoted with a “~” if the linear regression was not statistically significant. Conditions are abbreviated and color coded as follows: unpowered (NoPwr) (gray), NMM reflex Gains of 0.8, 1.2, 1.6, and 2.0 all with reflex Delay=10 ms (G0.8, G1.2, G1.6, and G2.0, respectively) (green), NMM reflex Delays of 10, 20, 30, and 40 ms all with a reflex Gain=1.2 (D10, D20, D30, D40, respectively) (blue), and a high-Gain-high-Delay condition (G2.0 D40) (black).

**Supplementary Figure 9. User’s relative muscle volume scaled (RMV) summed ankle muscle activity versus metabolic rate.** Measurements of users’ summed normalized electromyography (EMG) signals recorded from soleus + medial gastrocnemius + lateral gastrocnemius + tibialis anterior (SOL+MG+LG+TA) over a stride from heel strike (0%) to heel strike (100%) of the same leg are shown (A, B). Bar graphs are averages of the summed EMG signals over the stride (C and D). All measurements are averages across the study participants (N=9) in each condition with varying neuromuscular model (NMM) controller reflex Gain (green) in left panel (A, C) and Delay (blue) in the right panel (B, D). Linear regression between the change in metabolic rate versus the change in average RMV summed EMG with respect to the unpowered (NoPwr) condition (E). * denotes pairwise significant difference of p < .05. R^2^ value is denoted with a “~” if the linear regression was not statistically significant. Conditions are abbreviated and color coded as follows: unpowered (NoPwr)(gray), NMM reflex Gains of 0.8, 1.2, 1.6, and 2.0 all with reflex Delay=10 ms (G0.8, G1.2, G1.6, and G2.0, respectively) (green), NMM reflex Delays of 10, 20, 30, and 40 ms all with a reflex Gain=1.2 (D10, D20, D30, D40, respectively) (blue), and a high-Gain-high-Delay condition (G2.0 D40) (black).

**Supplementary Figure 10. Intersubject variability across measurements.** Measurements of users’ ankle angle (A), ankle velocity (B), exoskeleton torque (C), exoskeleton power (D), biological ankle moment (E), biological ankle power (F), soleus (SOL) electromyography (EMG) (G), medial gastrocnemius (MG) EMG (H), lateral gastrocnemius (LG) EMG (I), tibialis anterior (TA) EMG (J), and summed EMG (K) over a stride from heel strike (0%) to heel strike (100%) of the same leg are shown. Ankle plantarflexion is depicted as positive and dorsiflexion as negative for the angle, velocity, and moment/torque plots. EMG data were normalized per muscle per subject to the maximum EMG signal during the unpowered condition. All data were first averaged across many strides per participant and then across all participants (N=9) per condition. Shaded regions represent the average plus or minus one standard deviation. Time-series data for the unpowered (NoPwr) (gray), NMM reflex Gain of 0.8 with reflex Delay=10ms (G0.8 D10) (green), and a high-Gain-high-Delay condition (G2.0 D40) (black) are shown.
