# Supplementary figures and images for: Neuromechanics and Energetics of Walking With an Ankle Exoskeleton Using Neuromuscular-Model Based Control: A Parameter Study
Source: Front Bioeng Biotechnol. 2021 Apr 9;9:615358. doi: 10.3389/fbioe.2021.615358 (PMC8091965; doi:10.3389/fbioe.2021.615358)

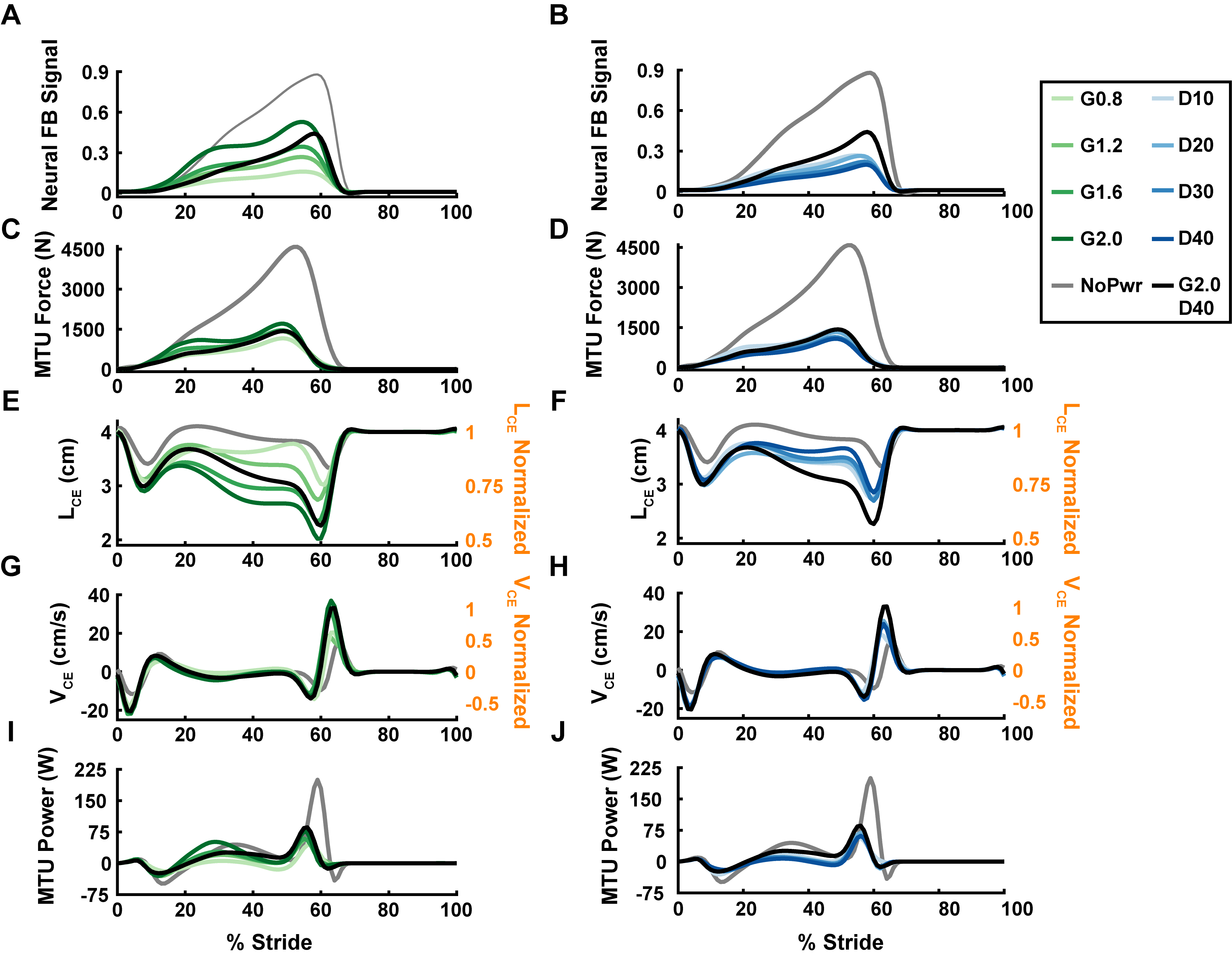

Supplement: Supplementary file 2 [file Data_Sheet_2.zip › SuppFig1_Frontiers_Review_V1.tif]

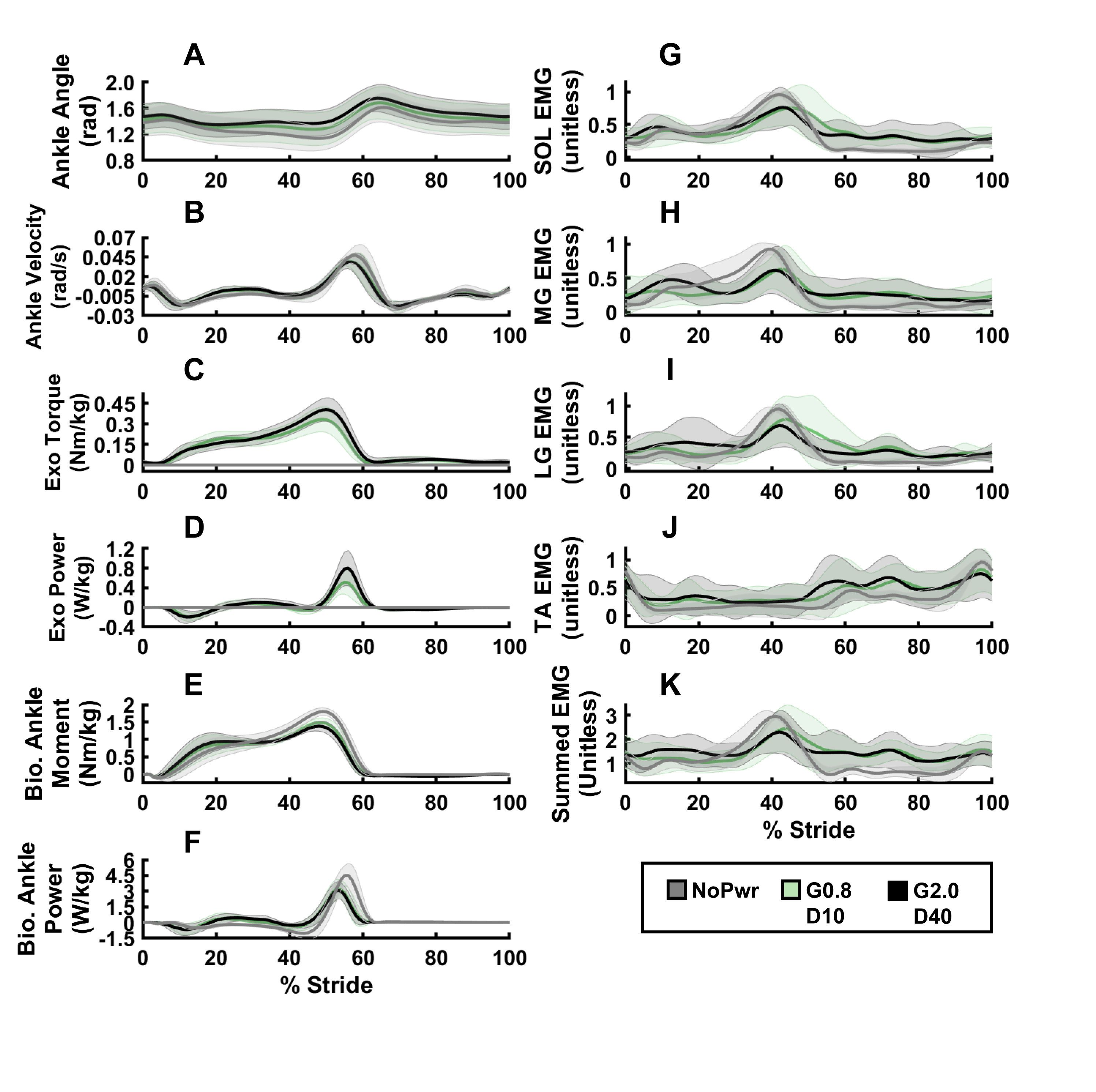

Supplement: Supplementary file 2 [file Data_Sheet_2.zip › SuppFig10_Frontiers_Frontiers_Review_V2.tif]

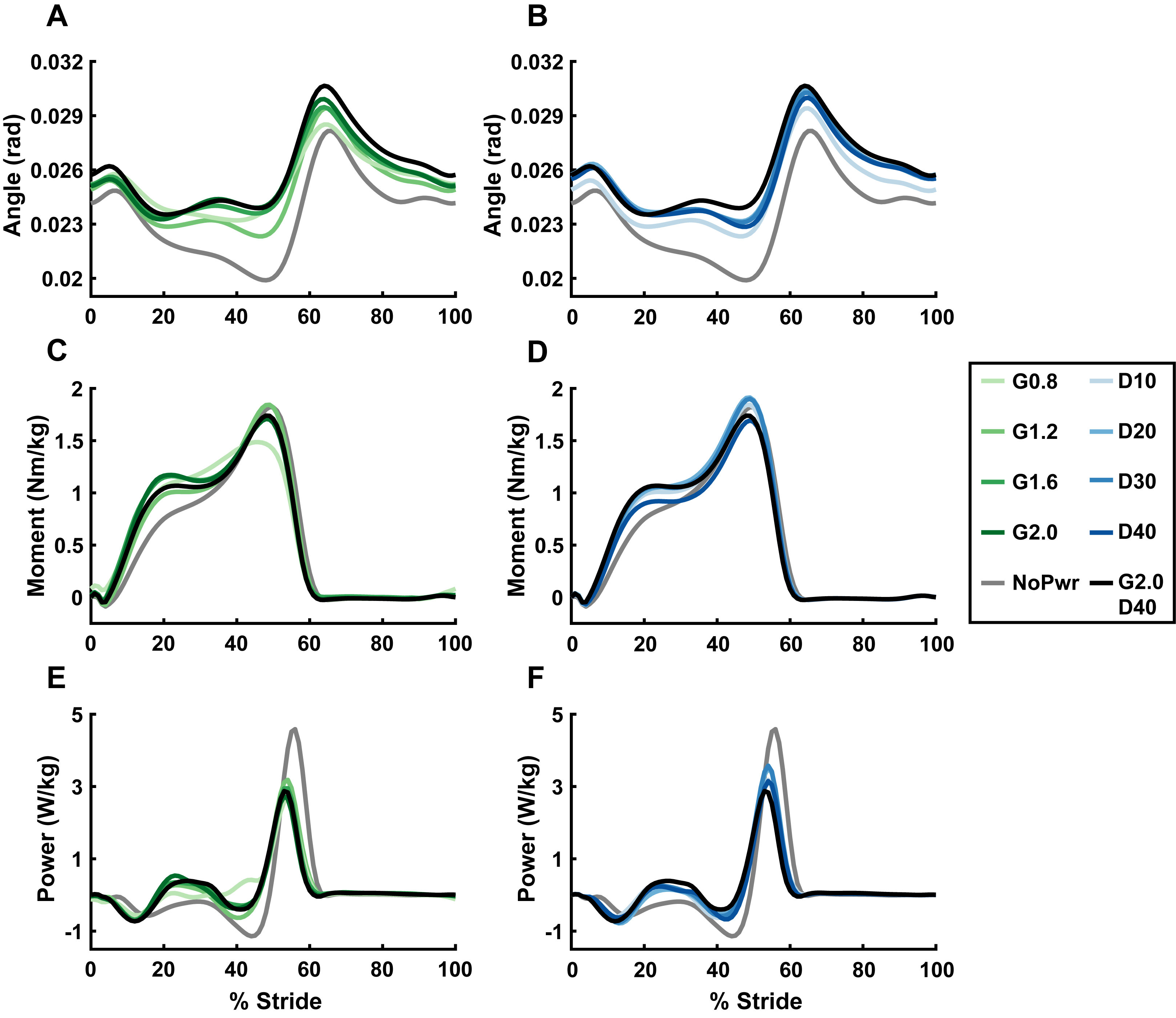

Supplement: Supplementary file 2 [file Data_Sheet_2.zip › SuppFig2_Frontiers_Review_V1.tif]

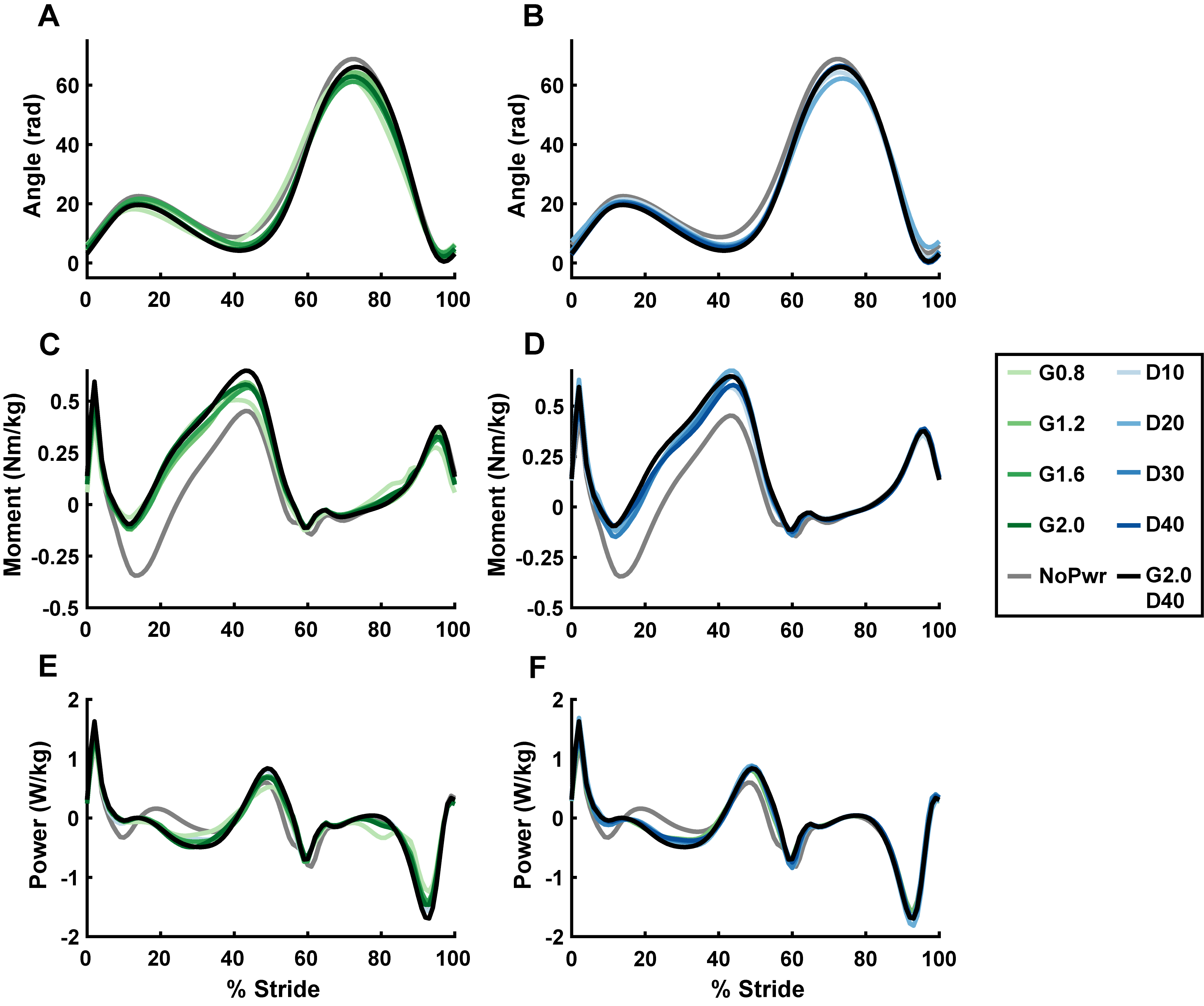

Supplement: Supplementary file 2 [file Data_Sheet_2.zip › SuppFig3_Frontiers_Review_V1.tif]

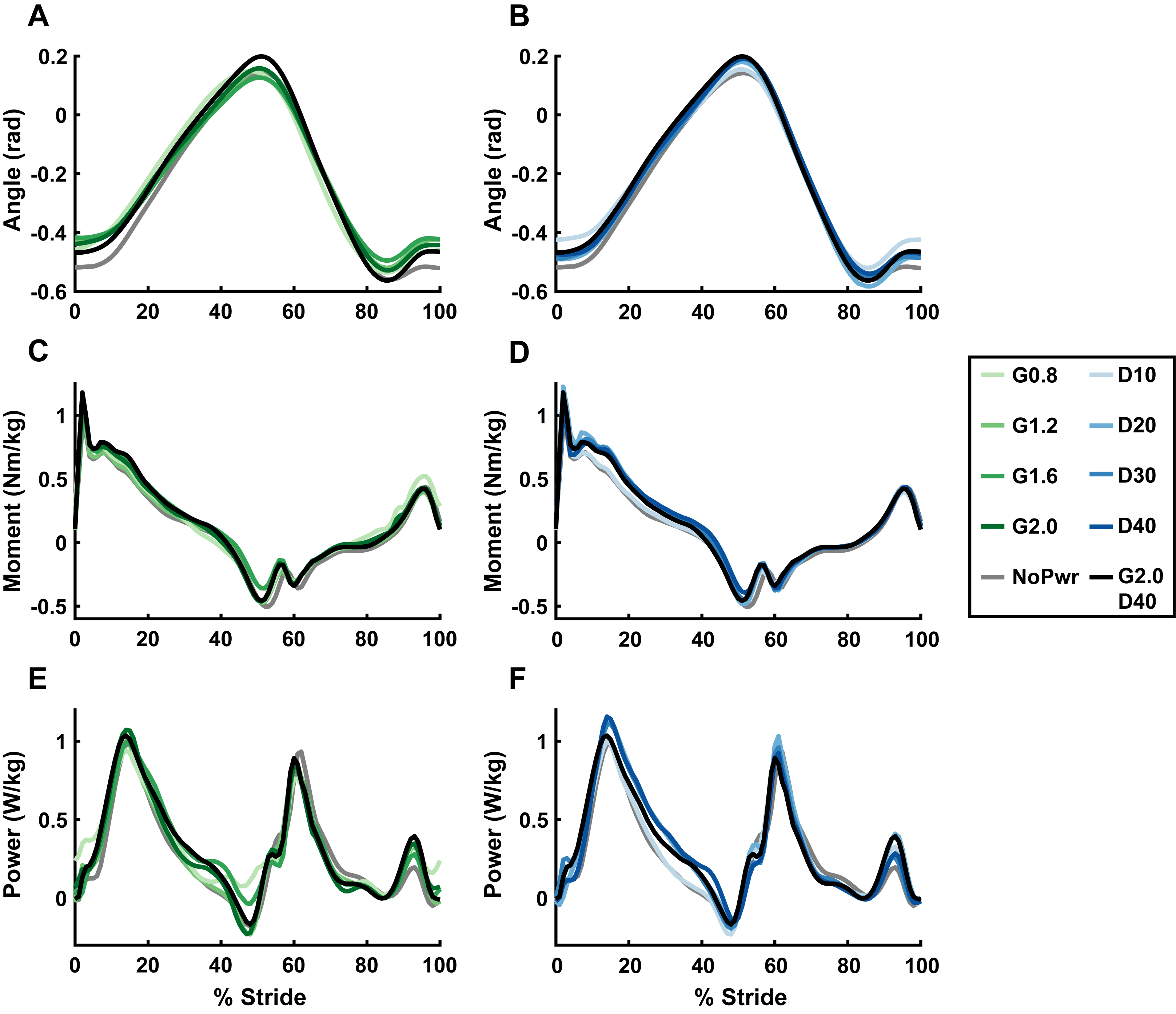

Supplement: Supplementary file 2 [file Data_Sheet_2.zip › SuppFig4_Frontiers_Review_V1.tif]

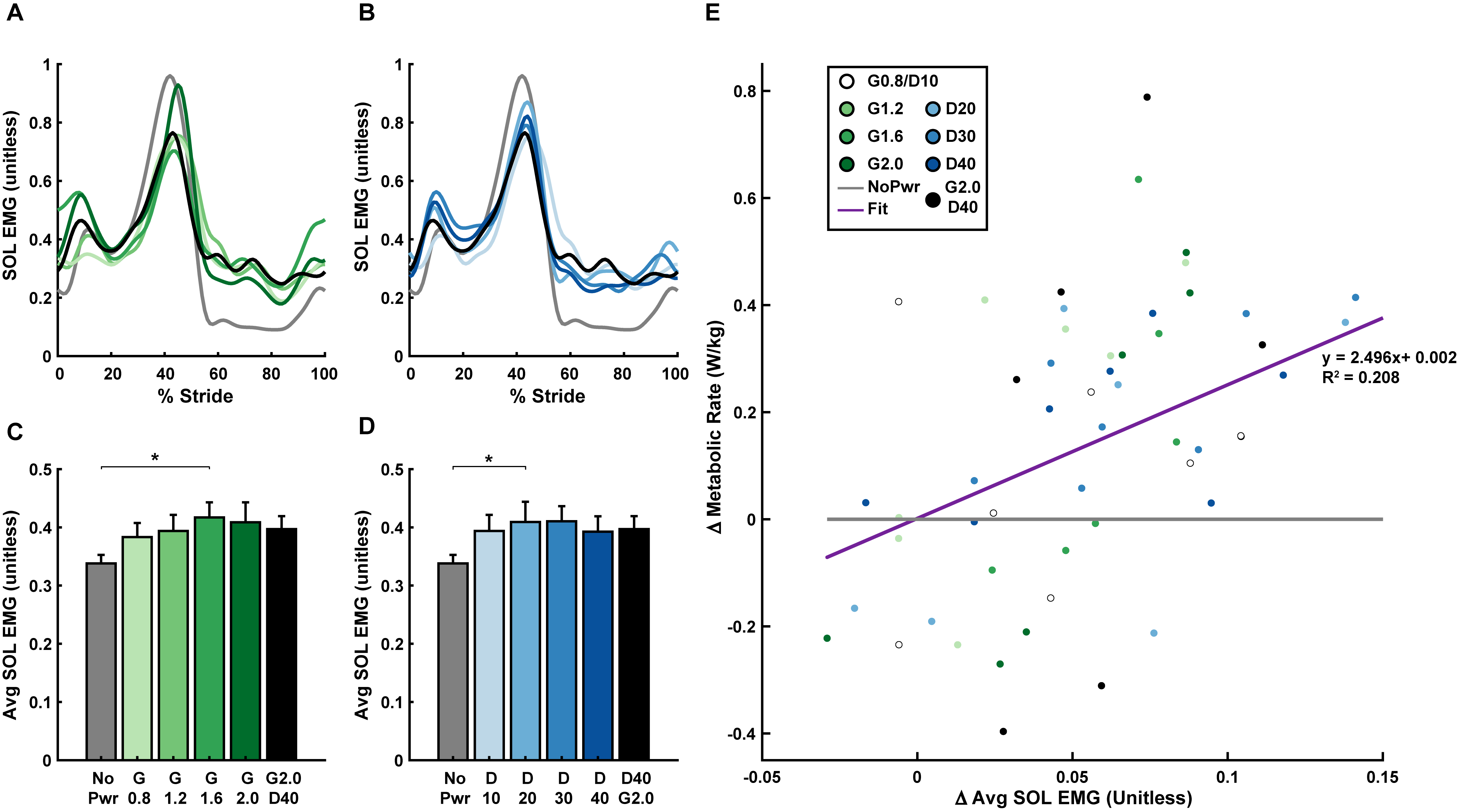

Supplement: Supplementary file 2 [file Data_Sheet_2.zip › SuppFig5_Frontiers_Review_V1.tif]

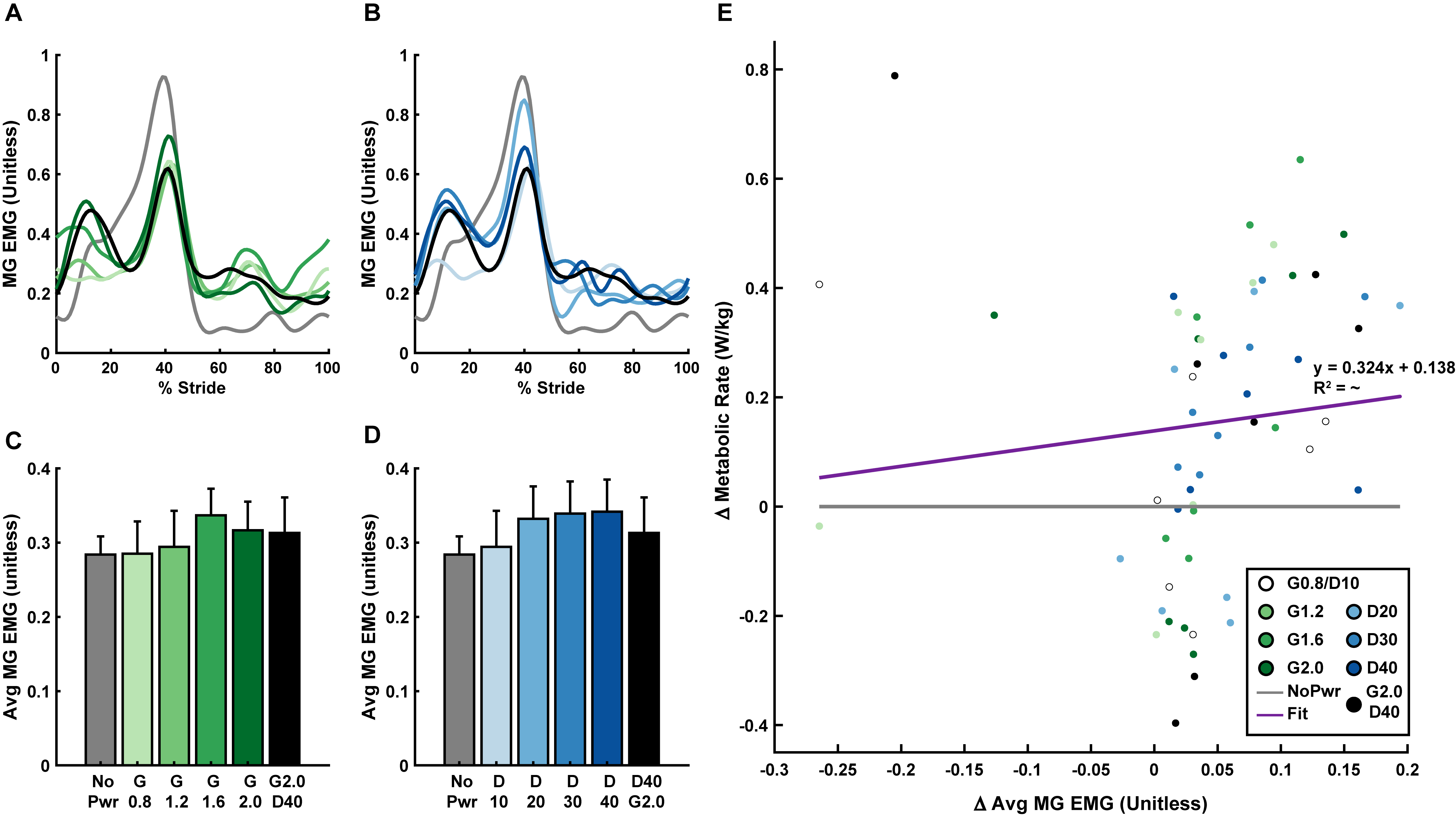

Supplement: Supplementary file 2 [file Data_Sheet_2.zip › SuppFig6_Frontiers_Review_V1.tif]

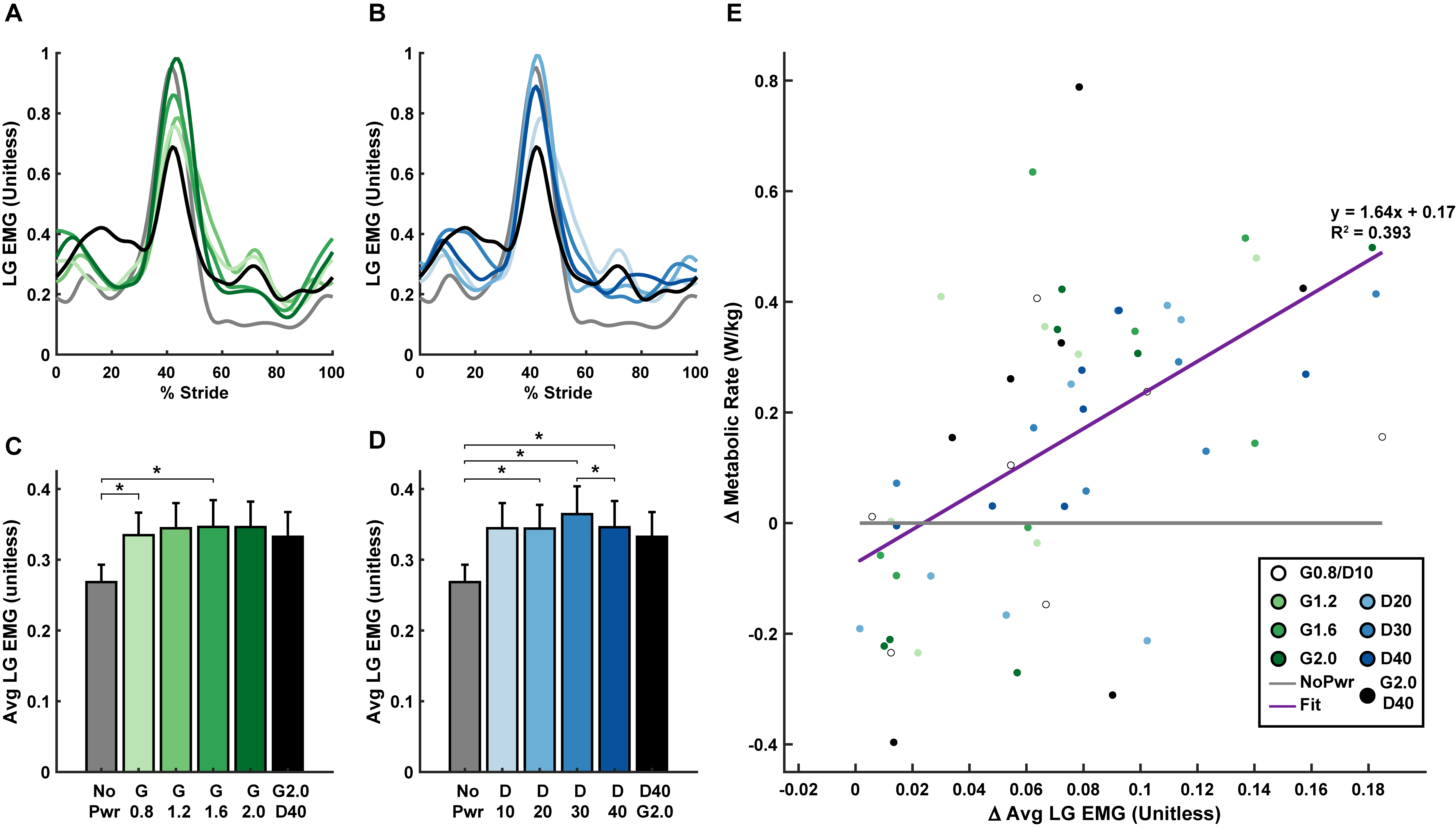

Supplement: Supplementary file 2 [file Data_Sheet_2.zip › SuppFig7_Frontiers_Review_V1.tif]

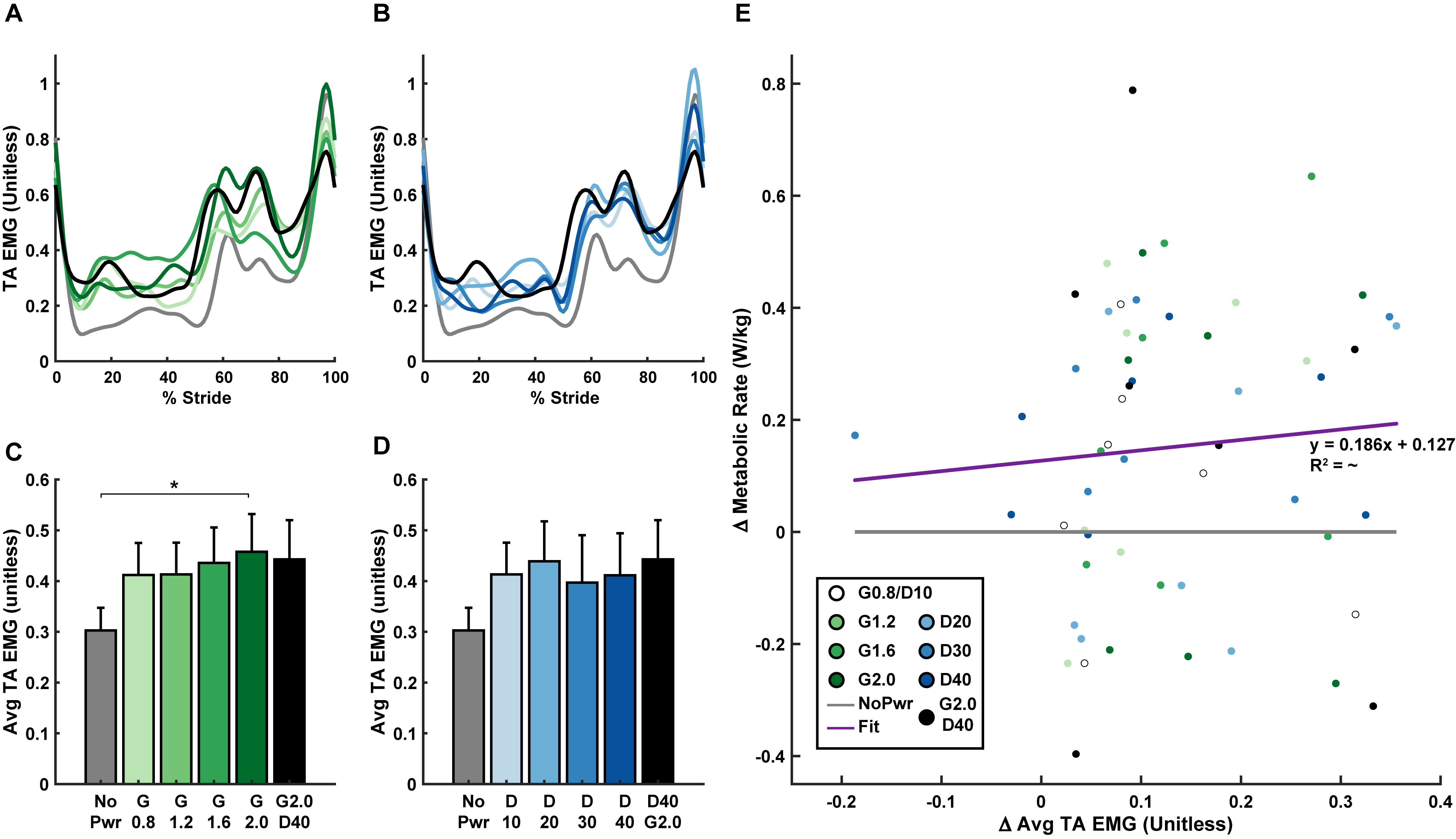

Supplement: Supplementary file 2 [file Data_Sheet_2.zip › SuppFig8_Frontiers_Review_V1.tif]

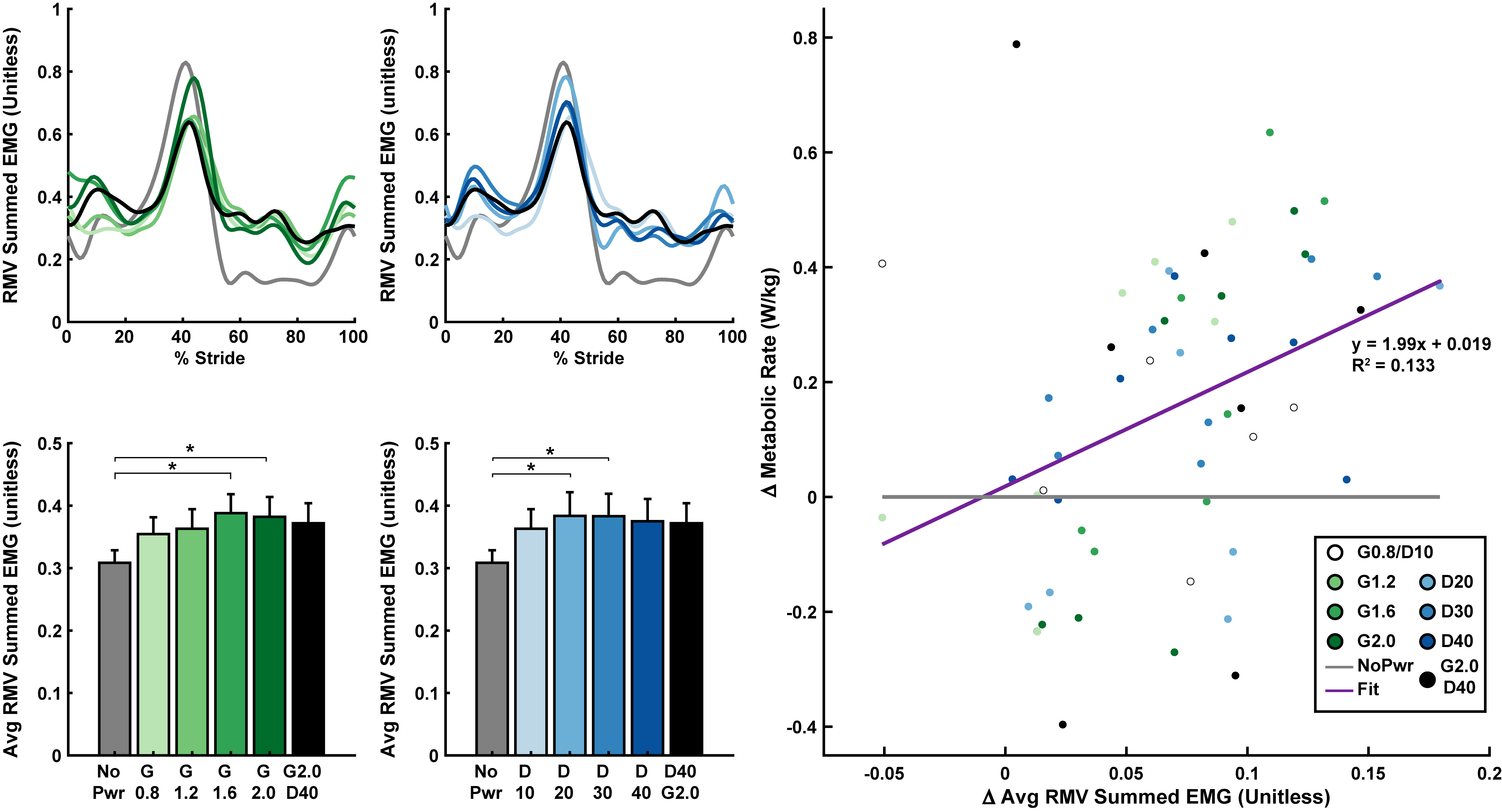

Supplement: Supplementary file 2 [file Data_Sheet_2.zip › SuppFig9_Frontiers_Frontiers_Review_V2.tif]
